# Can Information About Stiffness Perception be Inferred from Action Signals Using Models?

**DOI:** 10.1523/ENEURO.0495-23.2024

**Published:** 2024-12-12

**Authors:** Hanna Kossowsky Lev, Ilana Nisky

**Affiliations:** ^1^The Department of Biomedical Engineering, Ben Gurion University of the Negev, Be'er Sheva 8443944, Israel; ^2^The School of Brain Sciences and Cognition, Ben Gurion University of the Negev, Be'er Sheva 8443944, Israel

## Abstract

We use sensory feedback to form our perception, and control our movements and forces (actions). There is an ongoing debate about the relation between perception and action, with evidence in both directions. For example, there are cases in which perceptual illusions affect action signals and cases where they do not. However, even when they do, it is unknown if perceptual information can be inferred from action signals alone. To answer this question, we utilized a perceptual illusion created by artificial tactile skin stretch, which increases stiffness perception, and affects grip force. We used data recorded in a stiffness discrimination task in which participants compared pairs of virtual objects, comprised of force and artificial skin stretch and indicated which they perceived as stiffer. We explored if models could predict the participants’ perceptual responses, and the increase in stiffness perception caused by the skin stretch, solely from their recorded action signals. That is, with no information provided about the stimuli. We found that participants’ perceptual augmentation could be predicted to an extent from their action signals alone. We predicted the average augmentation effect across participants, and a general trend of increased predicted perceptual augmentation for increased real perceptual augmentation. These results indicate that at least some perceptual information is present in action signals. Furthermore, of the action signals examined, grip force was necessary for predicting the augmentation effect, and a motion signal (e.g., position) was needed for predicting human-like perception, shedding light on what information may be presented in the different signals.

## Significance Statement

We utilized artificial skin stretch and models to explore if participants’ actions are indicative of their perception, and found that aspects of perception can be predicted from action signals. Furthermore, our results shed light on which action signals are needed for predicting human-like perception, and which are indicative of the skin stretch augmentation effect. Beyond the scientific insights, this work could be used for the design of haptic feedback for force-displaying technologies. Trained models can be used to receive action signals and estimate the perceptual effect of different haptic stimuli. This can be advantageous, as it is commonly possible to record users’ action signals, however, the experiments required to measure perceptual effects are often long and require many repetitions.

## Introduction

We use haptic feedback to form our perception and control actions, such as our movements and forces. The literature is ambivalent about the association or dissociation between the processing of sensory information for perception and action, with evidence in each direction (Goodale and Milner, 1992; Goodale and Humphrey, 1998; Flanagan and Beltzner, 2000; Franz et al., 2000; Bruno, 2001; Haffenden et al., 2001; Ganel and Goodale, 2003; Post et al., 2003; Grandy and Westwood, 2006; Ganel and Goodale, 2014; Platkiewicz and Hayward, 2014; Leib et al., 2015; Bi et al., 2018b). Many studies use perceptual illusions to explore this question. For example, the Ebbinghaus illusion was originally shown to affect size perception, but not grip aperture before object grasping (Aglioti et al., 1995). Later evidence suggested that this illusion affects both perception and action, but to different degrees (Franz et al., 2001). The debate about the association or dissociation remains unresolved. In this paper, we examine a case where perception and action appear associated and ask whether action is indicative of perception in this case.

Previous works have used models to explain perception. For example, delays in force feedback affect stiffness perception, and (Pressman et al., 2006) modeled this using force and position information. Such models can also explain how altering an elastic object’s boundary affects stiffness perception (Pressman et al., 2011). Additionally, (Metzger et al., 2018; Kossowsky et al., 2021) studied stiffness perception when the force field stiffness varies between interactions. They found that participants formed their perception by computing a weighted average of the different stiffness levels. These works, however, included information about the stimuli (force or stiffness), whereas we aim to predict participants’ perception using only their actions. Several visual illusions were also shown to affect both size perception and grasping, with a correlation between the effects (Franz et al., 2001). However, they did not use participants’ actions to predict perceptual responses on a trial-by-trial basis, which, to our knowledge, remains unexplored. Additionally, these works did not employ machine learning models to learn perceptual information from action signals.

We explore the possibility of inferring perceptual information from action signals using a haptic illusion. Haptic feedback is comprised of two modalities – kinesthetic (sensed by Golgi tendon organs and muscle spindles) and tactile (sensed by cutaneous mechanoreceptors) (Kandel et al., 2000). One form of tactile feedback is skin stretch, caused by shear forces from friction between the fingerpad and an object. Devices can generate artificial skin stretch by moving a pin with a flat high-friction top against the skin (Witney et al., 2004; Prattichizzo et al., 2012; Quek et al., 2014). When applied with force feedback, artificial skin stretch creates the perceptual illusion of increased stiffness perception (Quek et al., 2014; Farajian et al., 2020). Artificial skin stretch in both the same, and the opposite, direction as force feedback increases stiffness perception, but the effect is stronger when the stretch is applied in the same direction (Farajian et al., 2023). In terms of action, artificial skin stretch affects grip force – the perpendicular force applied between the fingers and an object (Farajian et al., 2020). Hence, artificial skin stretch affects both stiffness perception and some aspects of action. Therefore, if perceptual information can be inferred from action signals, we could potentially predict the perceptual augmentation from action signals alone.

We used data recorded in a stiffness discrimination task in which participants interacted with pairs of virtual objects that applied force and artificial skin stretch and determined which they perceived as stiffer (Farajian et al., 2023). We designed models to receive action information as input and output participants’ predicted perception. We tested both simple models, which received metrics derived from action signals, and artificial neural networks, which can learn complex features, as well as nonlinear, and potentially temporal, relationships from the data. We assessed if our models could predict the increase in stiffness perception caused by the skin stretch. Beyond analyzing perceptual biases, we can also evaluate participants’ decision-making processes. As the difference between the two virtual objects’ stiffness levels increases, humans generally make fewer mistakes. We tested our models’ abilities to predict this behavior. By inputting different combinations of action (motion and grip force) signals, we studied their roles in predicting the perceptual augmentation and human-like perception. Additionally, we evaluated a model’s ability to predict the difference in the magnitude of the augmentation effect caused by skin stretch in the same, and in the opposite, direction as the force feedback.

## Materials and Methods

### General framework

**Figure 1.**
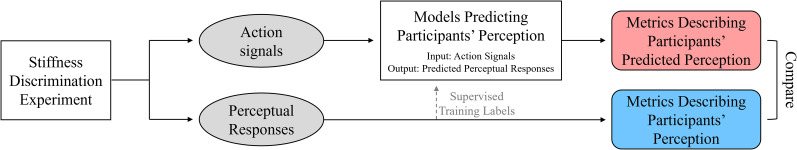
General framework. In a stiffness discrimination task composed of trials (Farajian et al., 2023), we recorded the participants’ action signals and perceptual responses. We used the perceptual responses to compute metrics describing the participants’ perception. Additionally, we designed models that received each trial’s action signals as input and outputted the predicted perceptual response of that trial. In relevant models, the participants’ real perceptual responses were used as true labels for training. We used the predicted perceptual responses to compute the same metrics describing the participants’ predicted perception. Lastly, we compared the predicted metrics to the real ones to assess the performance of the models.

We explored the possibility of predicting participants’ perception using their action signals. We used data we collected in a stiffness discrimination task (Farajian et al., 2023). The task was comprised of trials, in which participants interacted with two virtual objects and chose which of the two they perceived as stiffer. Throughout the experiment, we recorded signals describing participants’ actions (e.g., position and grip force) and logged their perceptual responses. Using the perceptual responses to all the trials, we computed metrics describing each participant’s stiffness perception. We used our models to predict the participants’ responses in each trial from their action signals. In relevant models, the participants’ real perceptual responses were used as true labels for training. Next, we computed the same metrics based on the models’ predictions and compared them to those of the participants. This general framework is illustrated in Figure 1. In the following, we briefly describe the experimental setup and protocol from (Farajian et al., 2023). Following that, we elaborate on the methods used for the prediction.

### Participants and ethics statement

Forty right-handed participants (19 females and 21 males, average age 24.58 ± 1.40) completed the experiment. The participants signed a consent form before beginning the experiment and were compensated for their participation, regardless of their success or completion of the experiment. The form and the experimental protocol were approved by the Human Subjects Research Committee of Ben-Gurion University of the Negev, Be’er-Sheva, Israel, approval number 1283-1, dated July 6th, 2015.

### Experimental setup

**Figure 2.**
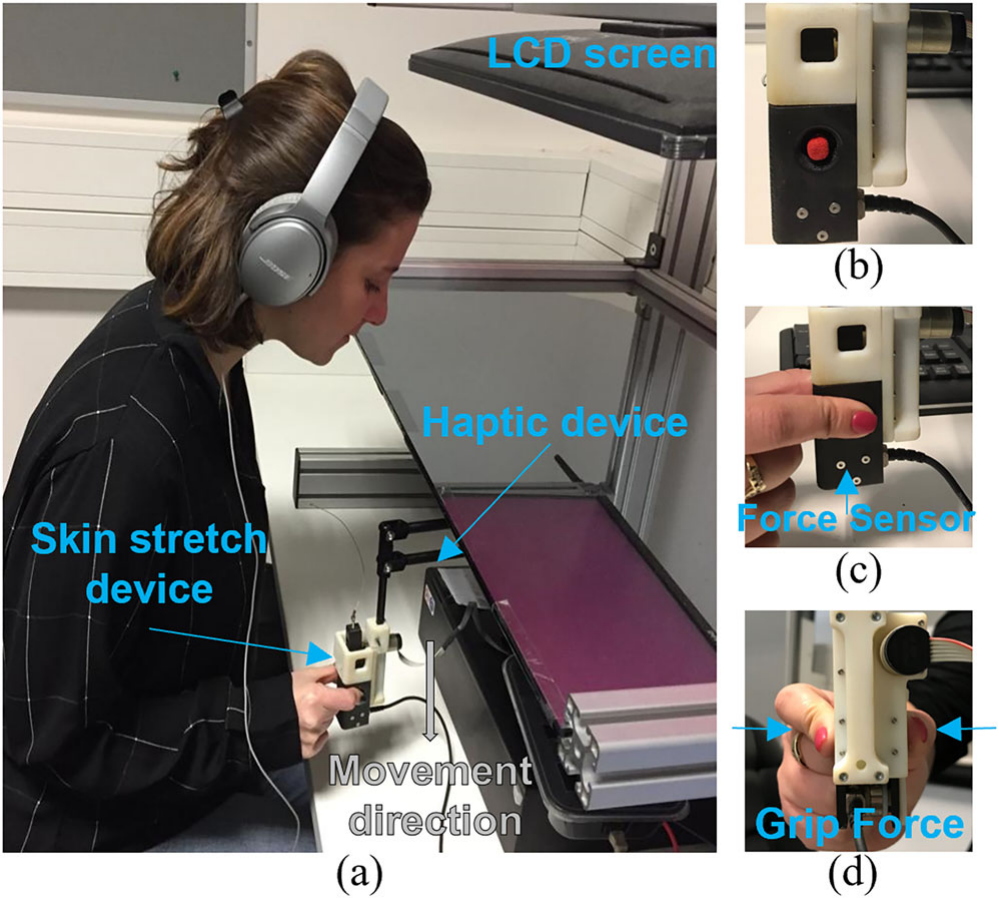
Experimental setup. ***a***, The participants sat in front of a virtual reality system and used a haptic device to make downward probing movements into virtual objects. ***b***, Side view of the skin stretch device, which was mounted on the end of the haptic device. The vertical movement of the tactors (red circles) created the artificial skin stretch. ***c***, Side view of the skin stretch device grasped by the participant, who placed her thumb and index finger on the tactors. ***d***, Back view of the skin stretch device, grasped by the participant. The grip force between the participant’s thumb and index finger was measured using a force sensor.

Participants interacted with a virtual environment using a PHANTOM ® Premium 1.5 haptic device (3D SYSTEMS, South Carolina, USA). They viewed the virtual environment through a semi-silvered mirror that blocked their view of their hand and showed the projection of an LCD screen placed above it (Fig. 2*a*). Participants used the index finger and thumb of their right hand to grasp the haptic device. This device conveyed kinesthetic information by generating a virtual elastic force field in the upward direction. The force was applied only when participants were in contact with the virtual object, which was defined to be the negative half of the vertical axis. The magnitude of the force was proportional to the penetration distance into the virtual object:

$$f=\left\{ \begin{array}{cc} -k\cdot y, & y<0,\\ 0, & y\geq 0, \end{array}\right.$$

where *k* [N/m] is the force field stiffness and *y* [m] is the penetration depth into the virtual object.

A 1-DOF skin stretch device was mounted on the end of the haptic device (Fig. 2*b–d*) and was used to induce artificial skin stretch on the fingerpads. The artificial skin stretch was created by two tactors (the red circles in Fig. 2*b*) that came into contact with the skin of the thumb and index finger on the palmer side and moved in the vertical direction. Similar to the force, the skin stretch stimulus was proportional to the penetration distance and was applied only when participants were in contact with the virtual object.

$$y_{\mathrm{tactor}}=\left\{ \begin{array}{cc} -g_{\mathrm{tactor}}\cdot y, & y<0,\\ 0, & y\geq 0, \end{array}\right.$$

where *g*_tactor_ [mm/m] is the tactor displacement gain and *y* [m] is the penetration depth into the virtual object.

A force sensor (ATI, Nano 17) was embedded in the skin stretch device to measure the grip force participants applied between their fingers. A detailed description of the experimental setup and skin stretch device can be found in Farajian et al. (2020).

### Experimental protocol

The experiment we conducted in Farajian et al. (2023) was a forced-choice stiffness discrimination experiment (Jones and Tan, 2013). The experiment was comprised of trials, in which participants made downward probing movements (Fig. 2*a*) into pairs of virtual objects (designated *standard* and *comparison*) that applied kinesthetic and tactile feedback, and reported which of the two felt stiffer. The order in which the two virtual objects were presented was chosen pseudo-randomly prior to the experiment. In each trial, one object was presented as blue using the LCD screen, and the other was red, also defined pseudo-randomly before the experiment. Participants interacted with each of the two virtual objects four consecutive times and then pressed a red or blue keyboard key to select which they perceived as stiffer.

The *comparison* virtual object applied only force feedback. The *standard* object applied force and, in some trials, also applied artificial skin stretch. Hence, there were two types of conditions: force conditions and artificial skin stretch conditions. In each trial, the *comparison* object was chosen to be one of 12 values equally spaced between 30 and 140 [N/m]. The *standard* stiffness level was 85 [N/m] in all the trials. The experiment was completed in two sessions, each of which was comprised of an identical force condition, and a skin stretch condition that differed between the sessions. In one session, the skin stretch was applied in the same (positive) direction as the force (*g*_tactor_ = 80 in Eq. 2), and in the other, in the opposite (negative) direction (*g*_tactor_ = −80). Each of the *standard*-*comparison* combinations in each condition were repeated eight times and the order in which they were presented was defined pseudo-randomly prior to the experiment. This led to a total of 96 trials per condition (192 trials per session). Additional details about the experimental protocols can be found in Farajian et al. (2023).

### Data

Throughout the experiment, we recorded several signals describing participants’ actions in each trial. We recorded the position and velocity in the vertical axis—the axis in which the participants’ movements were made. We also recorded the grip force participants applied between their fingers and the skin stretch device. We used these three signals and additionally calculated the acceleration numerically by differentiating the velocity according to the time. The signals were sampled at approximately 80 Hz. Furthermore, we recorded participants’ perceptual responses, which they logged using keyboard keys. Our goal in this work was to assess the possibility of predicting participants’ answers from their action signals.

The possibility of predicting perception from action signals can indicate that there is perceptual information present in the action signals. However, if we were to use trials with only force (and no artificial skin stretch), then the event of successful prediction would not necessarily indicate a relation between the participants’ perception and action signals. This is due to the fact that the *comparison* force field stiffness varied between trials. Additionally, the stiffness levels of the *standard* and *comparison* were not the same. Hence, we expect differences between the action signals that are related to the physics of the force field and not to the participants’ perception. For example, participants made deeper probing movements into less stiff force fields. While this may have been the result of perception, it could also have been caused by decreased resistance applied by the lower force field relative to the higher one. In order to eliminate this ambiguity, we included the artificial skin stretch trials. It has been well established that adding positive artificial skin stretch to force feedback increases the perceived stiffness (Quek et al., 2014; Farajian et al., 2020, 2021; Kossowsky et al., 2022; Farajian et al., 2023). However, the artificial skin stretch does not apply any forces that would impede the penetration into the virtual object. That is, artificial stretch is a stimulus that affects stiffness perception, without affecting the physics of the force field. Therefore, in addition to assessing if our models can predict the participants’ responses in the force condition, we investigated if participants’ responses in the artificial skin stretch condition, and the increase in stiffness perception caused by the stretch, can be predicted from the action signals. It is important to note that the action signals contain no information about the stimuli applied on the participants, enabling us to study if perceptual information can be learned solely from action signals.

#### Action signal preprocessing

**Figure 3.**
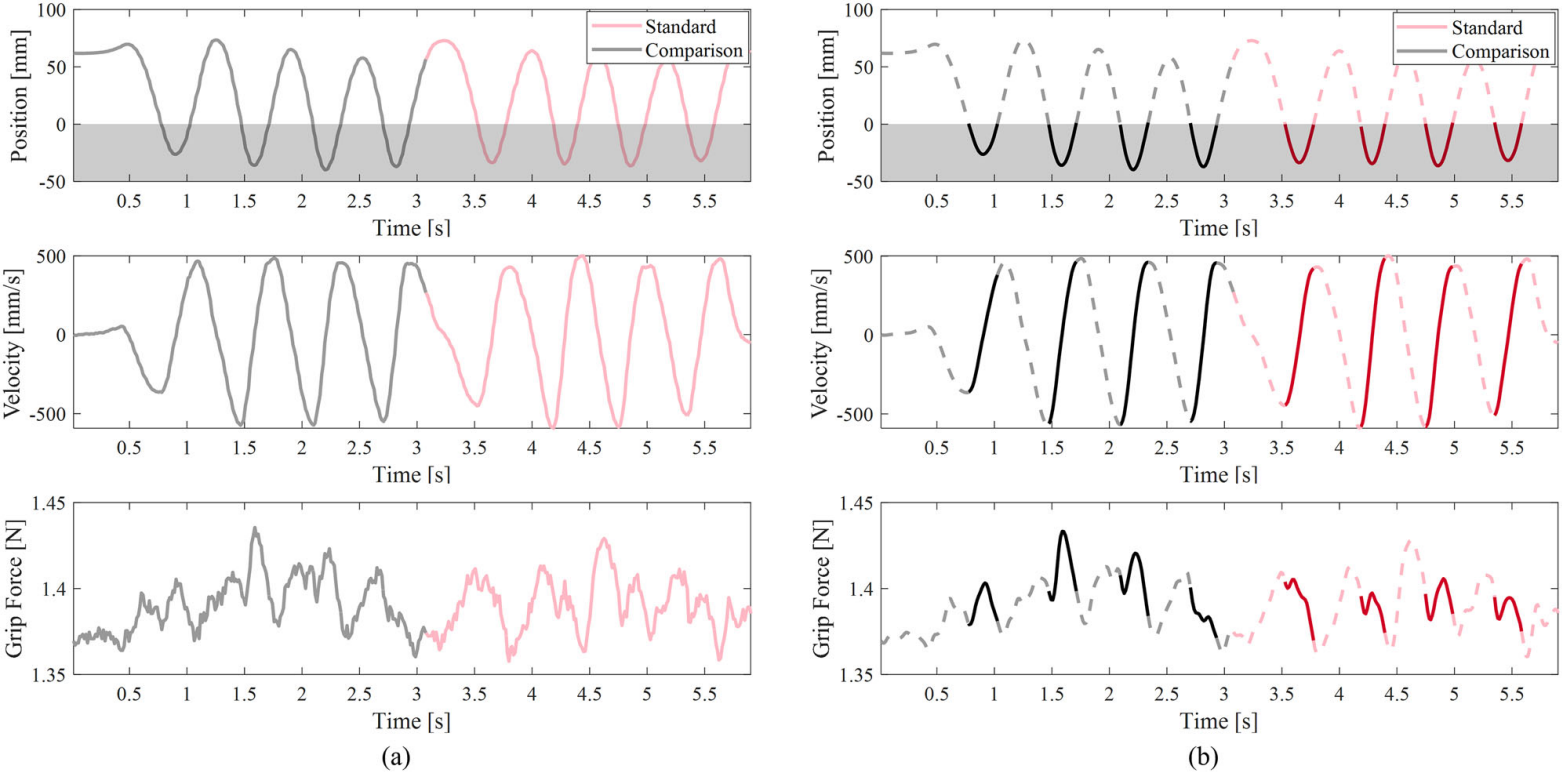
Signals for one trial. ***a***, Raw and ***b***, Filtered. The top graph shows the position, the second the velocity, and the bottom is the grip force. Gray indicates an interaction with the *comparison* object, and pink is the interaction with the *standard* object. The shaded gray area in the position signals indicates the portions of the signal in which participants interacted with the virtual objects (i.e., were in the negative half of the vertical axis). Only these portions of the signals were extracted and are shown using solid, darker lines in (*b*). The portions of the signal that were not used (participants were above the boundary of the object) are dashed in (*b*).

Figure 3*a* shows an example of each recorded action signal before the preprocessing. Figure 3*b* shows the three recorded signals after filtering. We filtered the data using second-order zero-phase Butterworth filters, applied using the filtfilt MATLAB function, resulting in fourth-order filters. The cutoff frequency was 9.63 Hz for the grip force signal, as in Farajian et al. (2020), and 8.02 Hz for the position, velocity and acceleration signals, similar to Kossowsky and Nisky (2022). Force and artificial skin stretch were applied only when participants were in contact with the virtual object (the negative half of the vertical axis). Hence, the signals also contained information from times in which participants were above the boundary of the objects. We therefore extracted the portions of the signals during which the participant had interacted with the virtual objects (Fig. 3*b*). Next, all the signals were interpolated to 150 samples for each the interaction with the *comparison* object, and the interaction with the *standard* object (that is, a total of 300 samples per trial). The last step was to normalize each of the four features by subtracting its mean and dividing it by the standard deviation (*z*-score normalization).

#### Perception and model evaluation

**Figure 4.**
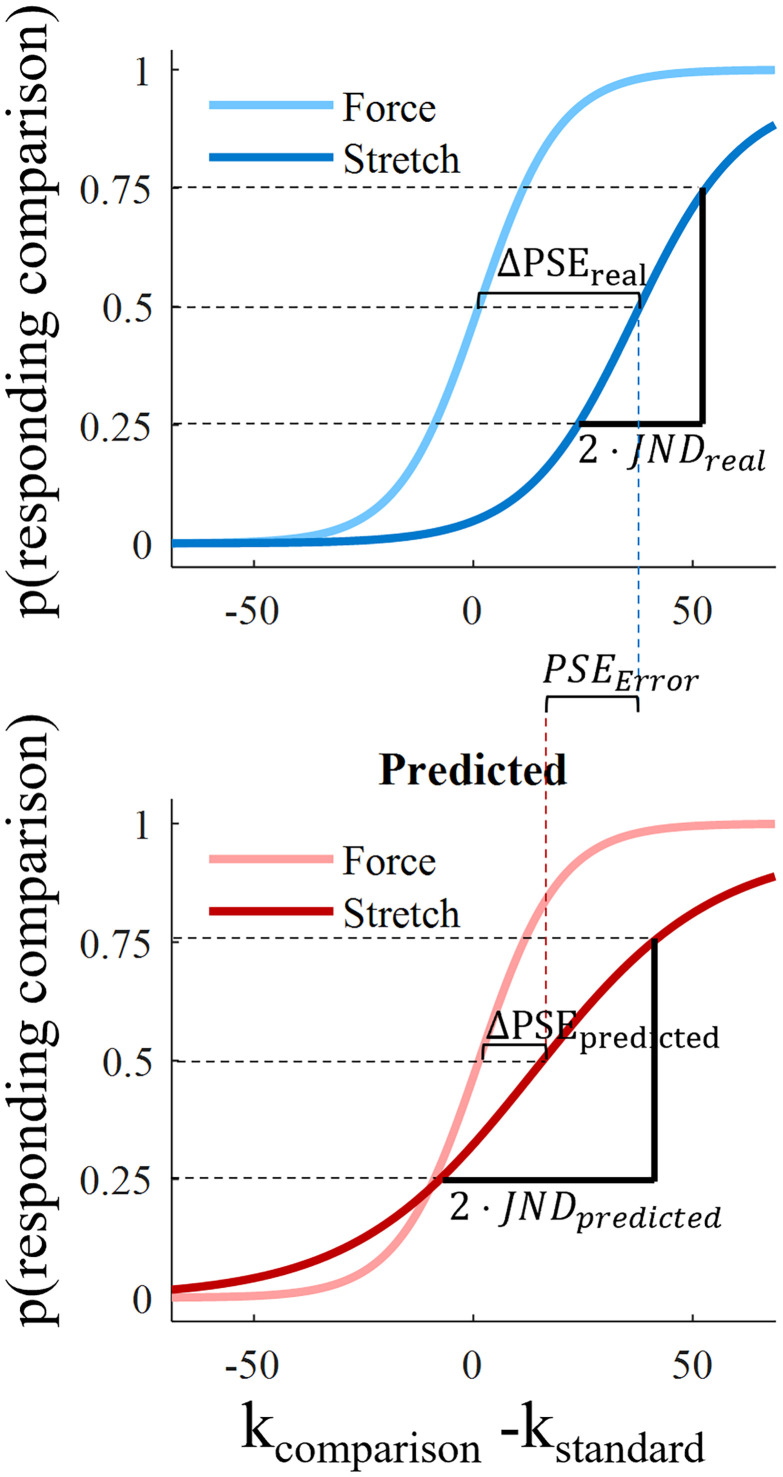
Synthetic psychometric curves. These are psychometric curves used for demonstration and explanation of the metrics, and are not the results of a specific participant. The pale-colored curves are for the force condition and the dark-colored curves are for the stretch condition. The abscissa is the difference between the stiffness levels of the two virtual objects, and the ordinate is the probability of choosing that the *comparison* object was stiffer. The blue curves show the hypothetical results of one participant, where the rightward shift of the dark blue curve indicates an increased stiffness perception due to the skin stretch. The red curves are the predicted results of the participant.

One approach that we used to evaluate our models is to assess their ability to predict participants’ perceptual answers in each trial and report the accuracy of the model. However, stiffness discrimination experiments open the possibility of quantifying additional aspects of perception. This is done by fitting psychometric curves to the participants’ responses (Jones and Tan, 2013). These curves quantify the probability of the participants’ responding that the *comparison* object felt stiffer than the *standard* as a function of the difference between the stiffness levels of the two virtual objects. This way, a single psychometric curve is fit to all the trials of each condition for each participant (Fig. 4). These curves can be more indicative of participants’ biases and sensitivities than the accuracy. For example, they can allow us to quantify the increase in stiffness perception (Wichmann and Hill, 2001).

We used the Psignifit toolbox 2.5.6 (Wichmann and Hill, 2001) to fit psychometric curves to participants’ responses. We then computed the point of subjective equality (PSE) and the just noticeable difference (JND) of each curve. The PSE is the stiffness level at which the probability of responding *comparison* is half and therefore measures the bias in the perceived stiffness. The rightward shift of the skin stretch psychometric curve (dark blue) in Figure 4 relative to the force (pale) one indicates an increase in the perceived stiffness of the *standard* object due to the stretch. The JND is half the difference between the stiffness levels corresponding to the 0.75 and 0.25 probabilities (illustrated on the dark red and blue curves in Fig. 4), and is an indication of the curve’s slope (Jones and Tan, 2013).

The PSE and JND define the shape of the curve (Wichmann and Hill, 2001). Therefore, to assess the ability of the models to predict the participants’ perception, we used these values to define several metrics. Figure 4 can be used to illustrate these metrics. The blue curves describe the perceptual results of a hypothetical participant. The pale blue curve is for the force condition and the dark blue curve is for the positive artificial skin stretch condition. The red curves are hypothetical curves created from a model’s predictions for the same participant. We focused on three metrics:

- Δ*PSE*: This is the difference between the PSE values in the stretch and force conditions (difference between the pale and dark curves in Fig. 4). Hence, this value quantifies the increase in stiffness perception caused by the artificial skin stretch in units of [N/m]. To assess the ability of the models to learn the perceptual augmentation from the action signals, we compare the Δ*PSE* of the real curves (blue) to that of the predicted curves (red). In the example curves (Fig. 4), the model predicted an increase in stiffness perception due to the stretch, but the predicted effect size was smaller than the real one. To analyze the results of all the participants, we perform a regression of the Δ*PSE*_*predicted*_ values against the Δ*PSE*_*real*_ values. The regression of an ideal model would have an intercept of zero and a slope of one.
- *PSE* *Error*: The Δ*PSE* examines the model’s ability to predict the shift between the two psychometric curves (the perceptual augmentation), but not its ability to predict the shape of each individual curve. Therefore, for each the force and stretch conditions, we compared the PSE of the real curve to that of the predicted curve. For example, to evaluate the prediction in the stretch condition, we compare the PSE of the dark blue curve in Figure 4 to that of the dark red one. This is shown using the blue and red vertical dashed lines. Similarly, for the force condition, we compare the PSE of the pale blue curve to that of the pale red curve. To compute the *PSE* *Error*, we subtract the predicted PSE from the real one. Hence, this metric results in two values—one for each of the two conditions. To examine the results of all the participants, we compute the average, and 95% confidence intervals, of the *PSE* *Error* values.
- *JND* *Error*: Similar to the *PSE* *Error*, however, using the JND. The *PSE* *Error* and *JND* *Error* assess a model’s ability to predict curves that represent participant-like perception. Even if the Δ*PSE* is predicted well, the predicted curves could both be shifted either to the right or the left, or with very high JND values, and these inaccuracies would not be reflected in the Δ*PSE*.

#### Dataset

Forty participants completed the experiment. Two of the participants were outliers: they exhibited a perceptual effect of the skin stretch that exceeded the measurable effect in this experiment. The highest *comparison* stiffness level was 140 [N/m]; therefore, if a participant has an increase in PSE beyond 55 [N/m], it is not measurable in this setting. Hence, the real PSE of the participant is not computable, and we therefore cannot assess the ability of models to predict it. We used the remaining 38 participants in our analyses. Note, that of the 38 participants, four exhibited a decrease in their PSE due to the skin stretch. These participants differ from the vast majority in Farajian et al. (2020), Farajian et al. (2023), Farajian et al. (2021), Kossowsky et al. (2022) and Quek et al. (2014) and were previously excluded from analyses for various reasons (Farajian et al., 2020). However, the larger number of participants in Farajian et al. (2023) indicates that this may be a consistent effect shown by a small portion of the population. Therefore, these participants (the negative effect participants) were included in all our analyses, however, we did take special note of them in our division of the data into train and test sets.

We created two datasets used in different stages of the work, both containing all 38 participants. As described earlier, there were two sessions in the experiment. One contained a positive (in the same direction as the force) stretch session, and the other had a negative (in the opposite direction) stretch session. We used both sessions’ force conditions for training our models (to increase the quantity of data). In the first part of our work, we used only the positive stretch trials and incorporated the negative stretch trials at a later stage. The choice to begin with the positive stretch session stemmed from the well-established effect of this stimuli, which has been shown in all of the aforementioned studies (Quek et al., 2014; Farajian et al., 2020, 2021; Kossowsky et al., 2022; Farajian et al., 2023). Additionally, the perceptual effect of the positive stretch has been shown to be larger and more consistent than that of the negative stretch. The negative stretch also increases stiffness perception on average across participants; however, this effect is smaller than that of the positive stretch, with larger between-participant variability (Farajian et al., 2023). We therefore aimed to first use the positive stretch session to test if the perceptual augmentation could be predicted from the action signals, and then examined the possibility of predicting the different perceptual effects in the different sessions.

- **Dataset 1:** This dataset contained the 192 trials from the positive stretch session (96 force, and 96 positive stretch, trials). Additionally, for the training stage only, we included the 96 force condition trials from the negative session to increase the amount of training data. These trials were excluded from the testing stage. Therefore, participants used in the training stage had 288 trials each, and those used for testing had 192 trials. The negative session force trials were excluded from testing as we compared the positive stretch condition to the force trials belonging to its session, as in Farajian et al. (2023). We note that there was no significant difference between the force trials of the two sessions (Farajian et al., 2023). Additionally, we tested the model’s performance on these force trials when using Dataset 2, such that they too were assessed in this work.
- **Dataset 2:** Here we included all the trials from the negative stretch session for both training and testing, leading to 384 trials per participant. We used this dataset to assess our models’ ability to predict the difference between the perceptual effects of the positive and the negative skin stretch.

We used *k*-fold validation, as in Wenliang and Seitz (2018), where we selected *k* = 10. We chose to use *k*-fold validation, rather than a single split into train and test to allow for comprehensive reporting of the prediction ability on the entirety of the participants. No model was ever trained on any of the trials of a participant on which it was tested. That is, for each fold, all of the trials of some participants were left for testing, and the trials of the remaining participants were used for training. As only four participants had a negative perceptual effect, we first split the 34 non-negative effect participants randomly into 10 folds. Following that, we randomly added the four negative effect participants into the folds. This was to prevent the case of networks from being tested on negative effect participants without having been trained on them. Despite this, we note that the negative effect participants had a very low representation in the data due to their small number. The models received the trials of all the training participants and aimed to predict their perceptual responses in each trial. In the testing stage, the trained models predicted the perceptual responses of the testing participants’ trials. Using these predicted responses, we created the predicted psychometric curves, from which we computed the predicted PSE and JND values. We then used each participants’ real and predicted PSE and JND values to compute our three metrics. We ran every model five times to ensure no effect of the random seed and present the average and standard deviation of our results across the runs.

### Models

#### Artificial neural networks

**Figure 5.**
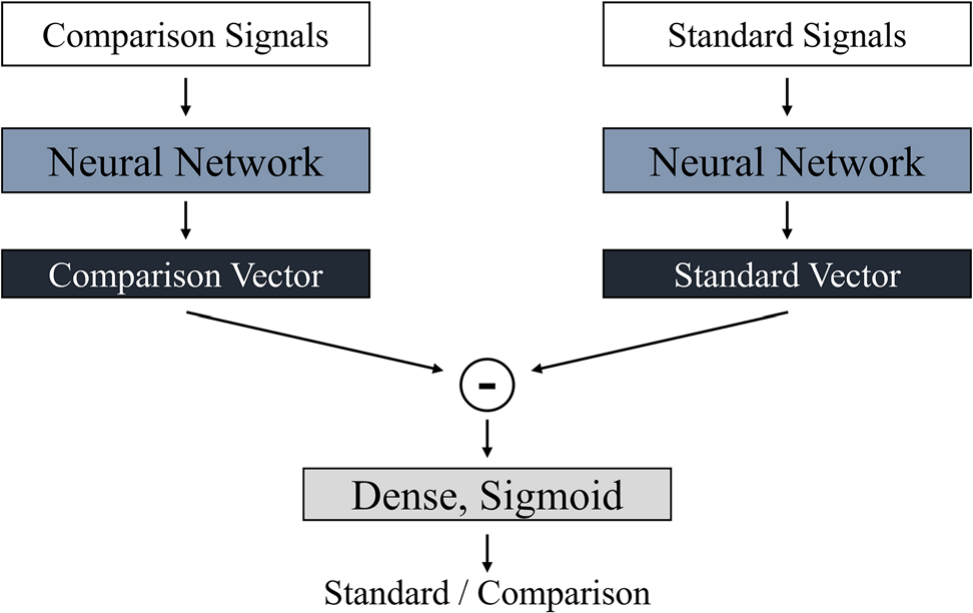
Artificial neural network. The *comparison* and *standard* signals were each inputted into identical neural networks. The network outputs were subtracted and inputted into a Dense layer with one neuron and a sigmoid activation. This layer classified the trial as 0 if the predicted response was *comparison*, and as 1 if *standard* was the predicted response.

We aimed to explore what artificial neural networks could learn about stiffness perception from participants’ action signals. We chose to use artificial neural networks due to their statistical power to learn complex patterns directly from data without requiring predefined feature engineering, as well as their ability to capture nonlinear, and temporal (when using Recurrent Neural Networks) patterns in the data. The participants’ preprocessed *comparison* and *standard* signals were each inputted into identical neural networks (Fig. 5). Hence, the inputs were vectors of 150 samples, containing four features per sample (position, velocity, acceleration, and grip force). The network outputted vectors of [128, 1], and the *comparison* vector was subtracted from the *standard* vector. This was then inputted into a Dense layer containing a single neuron and a sigmoid activation function, which outputted a value between zero and one. All outputs below 0.5 were classified as the response *comparison*, and those above 0.5 were *standard*. This two-stream network with subtracted outputs is similar to the general architecture in Wenliang and Seitz (2018).

The architecture of our network was a bidirectional long short term memory (LSTM) layer with 128 neurons, followed by a dropout layer with a probability of 0.4, batch normalization, and an additional bidirectional LSTM layer with 64 neurons. The second LSTM layer reduced the temporal information from 150 to one. We used LSTM layers (Hochreiter and Schmidhuber, 1997), as they are able to learn temporal relations due to a memory unit, and our inputs were temporal signals. We used dropout and batch normalization (Ioffe and Szegedy, 2015) to reduce over-fitting and stabilize the learning process. Our neural networks were trained for 50 epochs using the Adam optimizer (Kingma and Ba, 2014) and Binary Cross Entropy loss. We used a batch size of 512, and the data was randomly shuffled between each epoch. For the LSTM layers, we used L2 regularization, with a value of 0.001. The initial learning rate was set to 0.0001 and was decayed with every epoch according to *lr*_*i*_ = 0.95 * *lr*_*i*−1_, where *lr*_*i*_ is the learning rate in epoch *i*.

#### Simple models

In addition to assessing if artificial neural networks could predict the participants’ perceptual responses, we also tested if simple models using simple action metrics could be enough to perform this prediction. The goal of these models was to examine if simple and explainable metrics are indicative of the participants’ perception. We examined four deterministic models, which required no training, and one classifier:

- **Maximum Penetration Model:** We computed the maximum depth to which the participant penetrated into each the *standard* and *comparison* virtual objects in each trial. The model assumes that the maximum penetration will be smaller for objects perceived as stiffer. Therefore, the model chooses the object with the higher penetration as the less stiff one in each trial. This is similar to one of the models for stiffness perception suggested in Pressman et al. (2006).
- **Maximum Velocity Model:** We computed the maximum velocity from the participants’ interactions with each the *standard* and *comparison* virtual objects in each trial. The model assumes that participants will move more slowly in virtual objects they perceive as stiffer. Therefore, the model chooses the object with the higher maximum velocity as the less stiff one in each trial.
- **Average Velocity Model:** This model is similar to the Maximum Velocity Model, however, uses the average velocity instead.
- **Maximum Grip Force Model:** We computed the maximum grip forces from the participant’s interactions with each the *standard* and *comparison* virtual objects in each trial. The model assumes that participants will apply higher levels of grip force when interacting with virtual objects they perceive as stiffer. This is based on the fact that grip force has been shown to be tightly coupled to the load force (Flanagan et al., 1993; Flanagan and Wing, 1993). Therefore, the model chooses the object with the higher maximum grip force as the stiffer one in each trial.
- **Logistic Regression:** It is possible that a learned combination of the described four metrics could lead to better results than deterministic models on each metric alone. We therefore trained a logistic regression model to predict participants’ perceptual responses in each trial using these four metrics.

### Exploration questions

We explored what aspects of stiffness perception our models could infer from action signals alone. Specifically, we aimed to answer the following:

1. Can artificial neural networks predict stiffness perception from action signals?
  We used Dataset 1 and assessed the ability of networks to predict participants’ perceptual responses from their action signals. We evaluated the ability to predict the perceptual augmentation caused by the artificial skin stretch and the shapes of the participants’ psychometric curves. We initially used only the positive stretch session, as the perceptual augmentation caused by the positive stretch has been well established (Quek et al., 2014; Farajian et al., 2020, 2021; Kossowsky et al., 2022; Farajian et al., 2023), and has been shown to be larger and more consistent than that created by the negative stretch (Farajian et al., 2023). Therefore, we first examine if this effect can be predicted from action signals before continuing further into the study of the different effects that may be able to be predicted.
2. Can simple models predict stiffness perception from action metrics?
  We continued with Dataset 1 and tested the ability of our simple models to predict the perceptual augmentation caused by the artificial skin stretch and the shapes of participants’ psychometric curves. We examined the simple models to assess if these simple and explainable metrics are sufficient to predict the perceptual illusion. In the event that they are not sufficient, the need for neural networks is explained, and it indicates that the temporal relation in the signals may contain important information.
3. What is the contribution of each action signal to the prediction?
  Using Dataset 1, we removed different action signals in turn. We assessed the effect of different combinations of the four action signals on the network’s performance using our three evaluation metrics.
4. Can the network learn the difference in the magnitudes of the perceptual effects caused by positive and negative artificial skin stretch?
  We used Dataset 2 to test the network’s ability to predict the trend observed in Farajian et al. (2023); both positive and negative stretch led to an overestimation of stiffness, but that caused by the positive stretch was generally larger than that caused by the negative stretch.

### Code accessibility

The code and data described in the manuscript are available: https://github.com/Bio-Medical-Robotics-BGU/Exploring-Perception-and-Action.

## Results

### Can artificial neural networks predict stiffness perception from action signals?

**Figure 6.**
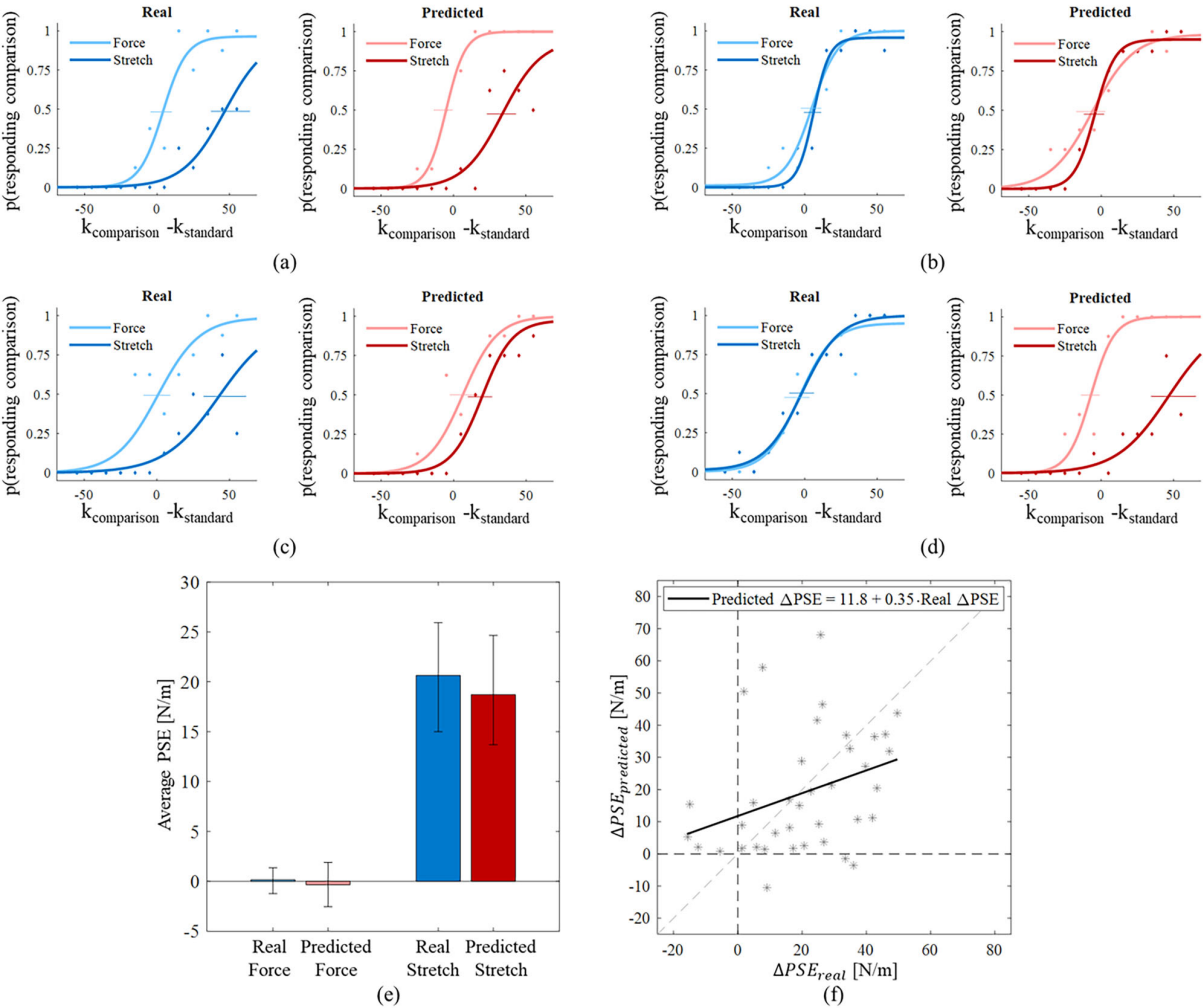
Neural network results. ***a–d***, Real and predicted psychometric curves of four participants. The blue curves were created using the participants’ real responses, and the red curves were created using the network’s predictions. The abscissa is the difference between the stiffness levels of the two virtual objects, and the ordinate is the probability of choosing that the *comparison* object was stiffer. The pale curves represent the force condition, and the dark curves are the stretch condition. ***a–b***, Participants predicted well by the network. ***c–d***, Participants whose perceptual effects were not predicted well. ***e***, The real (blue shades) and predicted (red shades) average PSE of all 38 participants for the force and stretch conditions. The bars show the average effects, and the black error bars are the 95% confidence intervals. ***f***, The results of all 38 participants. The gray stars show each participant’s predicted Δ*PSE* relative to their real one. The black line shows the regression of the predicted Δ*PSE*s against the real Δ*PSE*s. The dashed black lines show zero on the abscissa and ordinate. The gray dashed line is the ideal model, that is, a model with an intercept of zero and a slope of one.

To assess the performance of the networks, we first computed participants’ real psychometric curves. Next, based on the network’s predictions, we computed each participant’s predicted psychometric curves. Figure 6*a–b* show examples of participants whose psychometric curves were predicted well by the network. Figure 6*a* shows a participant whose stiffness perception was increased by the artificial skin stretch, and whose perceptual augmentation was captured well by the network. In Figure 6*b*, on the other hand, the participant’s perception was not increased by the stretch, and this lack of effect was predicted by the network. However, there were also participants whose effect was predicted less well by the network (Fig. 6*c–d*).

To analyze the results of all the participants, we assessed the ability of the network to predict the average effect across participants (Fig. 6*e*). Comparing the pale blue and red bars demonstrates the ability of the network to predict a near-zero effect in the force condition, and a similar average augmentation effect in the stretch condition (compare dark red and blue bars). That is, we obtained low *PSE* *Error*s in both conditions.

Next, we compared the Δ*PSE*_*real*_ for each participant (the difference between the pale and dark blue curves in units of [N/m]) to the Δ*PSE*_*predicted*_ (the difference between the pale and dark red curves) (Fig. 6*f*). We computed the regression of the Δ*PSE*_*predicted*_ against the Δ*PSE*_*real*_. Each star represents a participant, and the regression line is shown in black. By examining the data points (stars), we can see a general trend of increased predicted augmentation for increased real augmentation. These results show that the network can predict the perceptual augmentation caused by the stretch to an extent, and indicates that the stretch may cause the participants’ action signals to resemble those of interactions with stiffer objects. However, it is important to note that the regression is far from perfect, and the network cannot predict the precise size of the perceptual augmentation for each participant. Furthermore, the regression was not significant, with an average *p*-value of 0.06 across the five runs. This demonstrates the ability of the network to predict a general trend, but not the actual effect size for each participant.

The full results of the network are shown in Table 2. The accuracy of the network was ∼78%, which is well above chance level (50%). In addition to the accuracy and regression parameters, we examined the ability to predict psychometric curves of similar shape to those of the participants using the *PSE* *Error* and *JND* *Error* (Table 2). As shown, the network was able to predict curves of similar shapes to those of the participants.

**Figure 7.**
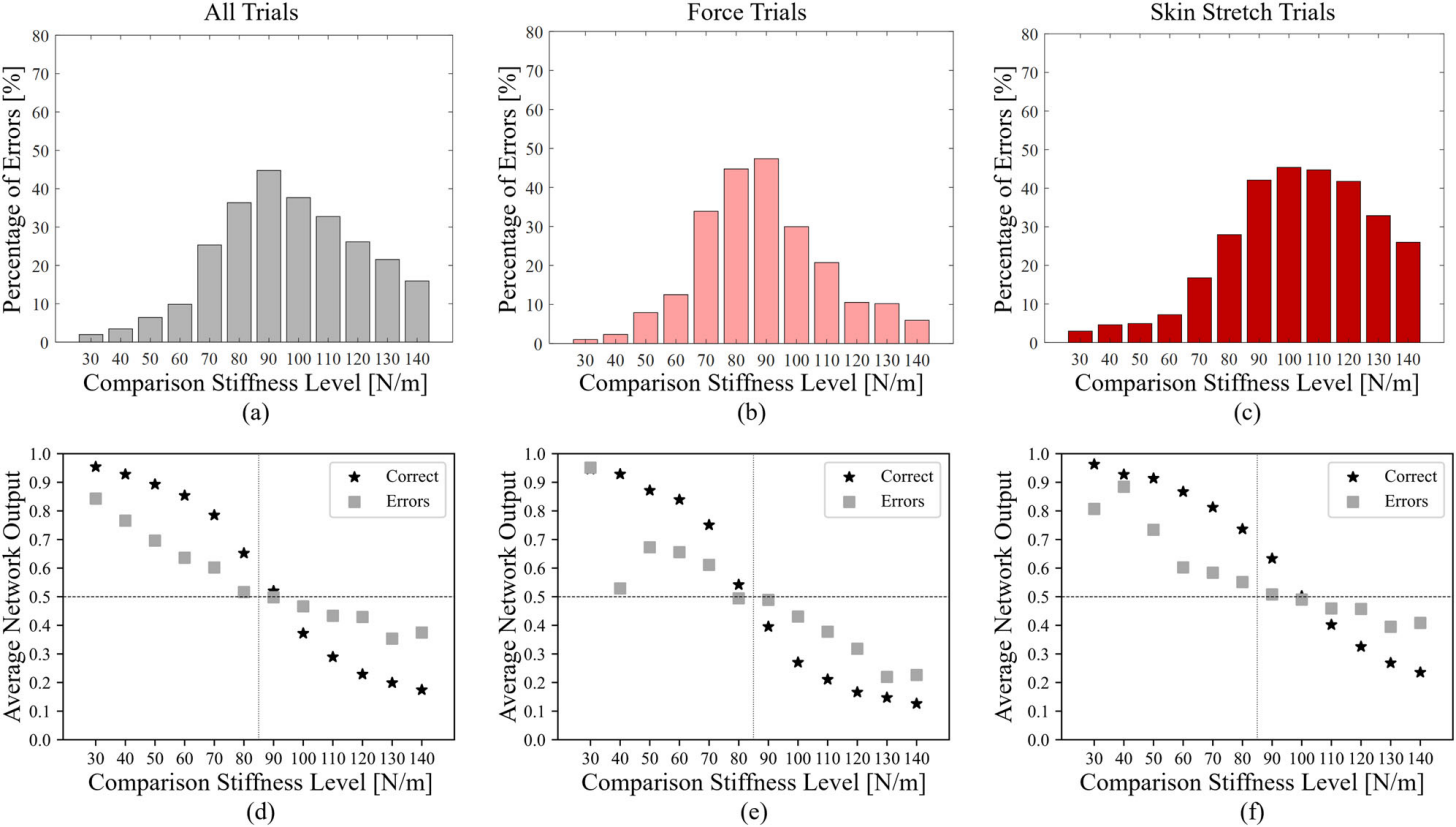
Errors and Certainty. ***a-c***, Network errors relative to the participants’ perceptual responses as a function of the *comparison* stiffness level for one of the five runs. The percentage of errors for each stiffness level was computed out of the total number of trials with that stiffness level in the given condition. ***a***, All the trials together. ***b***, The force trials alone. ***c***, The artificial skin stretch trials alone. ***d–f***, The average outputs of the network as a function of the *comparison* stiffness level for one of the five runs. This reflects the certainty, or loss value, of the network for each *comparison* level. The average outputs for correct predictions are indicated with black stars, and for mistaken predictions, with gray squares. In trials with the response *standard*, outputs closer to 1 reflect better performance, whereas for trials in which the response was *comparison*, outputs closer to 0 are superior. ***d***, All the trials together. ***e***, The force trials alone. ***f***, The artificial skin stretch trials alone.

We also analyzed the distribution of errors in the network’s predictions relative to the participants’ responses (the true labels) for the different *comparison* stiffness levels (Fig. 7*a–c*). An examination of Figure 7*c* (stretch trials only) reveals an asymmetrical distribution, with a larger percentage of errors for higher *comparison* stiffness levels. This can be due to the artificial skin stretch. The fact that the network could predict a general trend of increased stiffness perception due to the stretch indicates that participants’ action signals may resemble those of interactions with higher stiffness levels. Therefore, the skin stretch may cause the *standard* signals to resemble those of interactions with higher stiffness values than 85 [N/m] (the *standard* stiffness level). Therefore, for *comparison* stiffness levels below 85 [N/m], the skin stretch could increase the chance that the network chose *standard* as stiffer, which is likely the same response as the participant, as the skin stretch increased the perceived stiffness. For *comparison* stiffness levels higher than 85 [N/m], there may be more errors due to the action signal not reflecting the precise perceptual increase experienced by the participant. This can cause the network either to wrongly predict that participants perceived the *standard* object as stiffer when they did not, or vice versa. Figure 7*b* shows the force-only trials, in which a more symmetrical distribution of the errors can be observed, as expected. Figure 7*a* shows all the trials together, and is, therefore, a combination of the two described distributions of errors. On the one hand, the distribution is asymmetrical due to the influence of the stretch trials. On the other hand, there is a peak around 85 [N/m], due to the force trials.

Lastly, we examined the certainty of the network in its predictions as a function of the *comparison* stiffness level. The sigmoid activation function outputted values ranging between zero and one. All values below 0.5 were classified as zero (*comparison*), and those above 0.5 were one (*standard*). Therefore, for a true label of zero, values closer to zero would yield a lower loss. For a true label of one, values closer to one are strived for. Figure 7*d* shows the certainty of the network for all the trials, Figure 7*e* for the force trials alone, and Figure 7*f* is the stretch trials alone. We separated between the cases of correct (black stars) and mistaken (gray squares) predictions. As shown, the highest levels of uncertainty are observed for *comparison* levels close to 85 [N/m], and as the *comparison* stiffness level moves further from this value, the certainty increases. Additionally, Figure 7*d–f* shows that the certainty of the network in cases of correct prediction is generally better than the certainty for the cases of mistaken prediction.

### Can simple models predict stiffness perception from action metrics?

Before continuing with the study of what neural networks can learn about stiffness perception, we wondered if a simpler model could be sufficient. We therefore computed metrics from the participants’ action signals and assessed their ability to predict participants’ perceptual responses using our five simple models. Four of the simple models were deterministic, and the logistic regression model was trained using *k*-fold validation (*k* = 10), similar to the artificial neural network.

**Figure 8.**
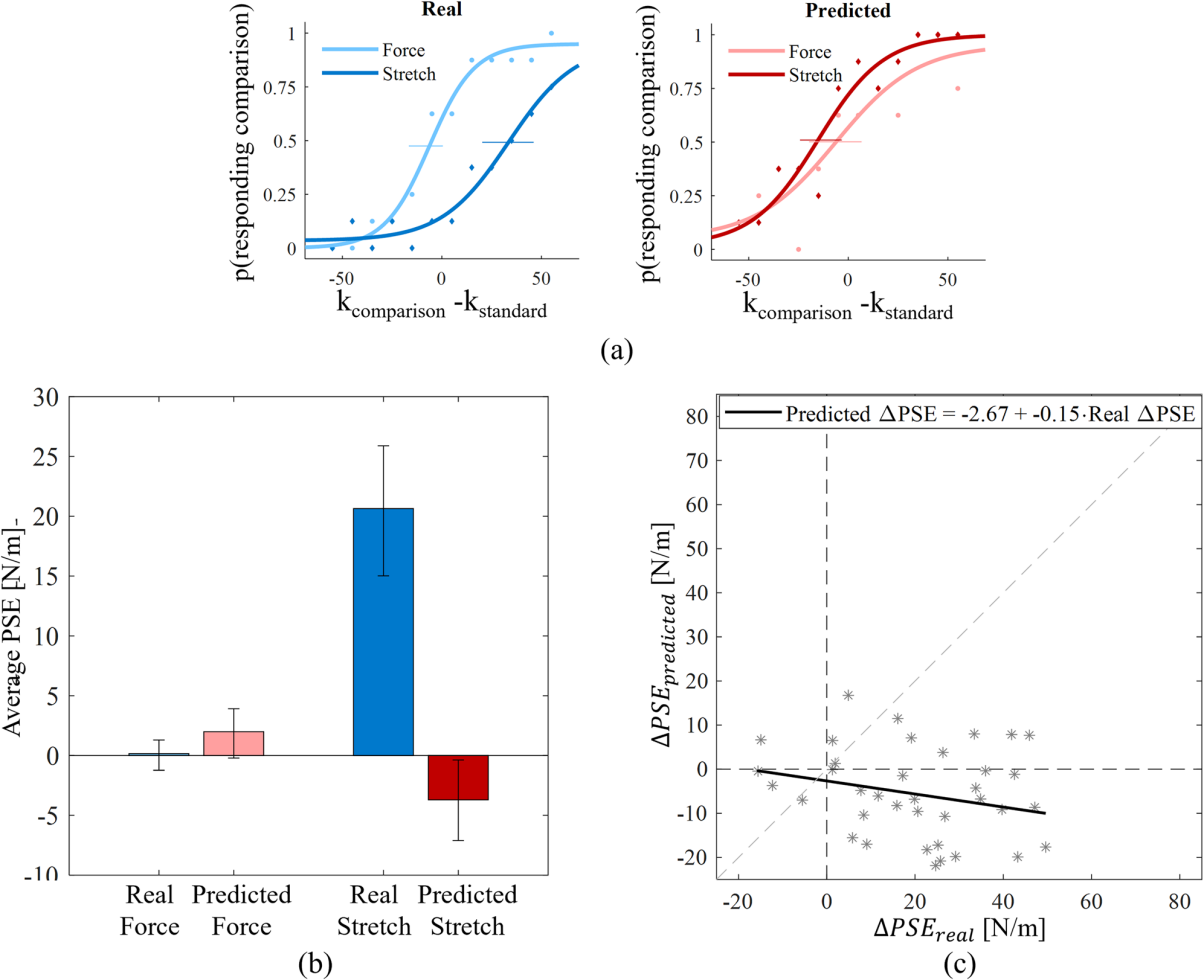
Maximum penetration model results. ***a***, Real and predicted psychometric curves of one of the participants. The blue curves are those created using the participant’s real responses, whereas the red curves were created using the predictions of the *Maximum Penetration Model*. In these graphs, the abscissa is the difference between the stiffness levels of the two virtual objects, and the ordinate is the probability of choosing that the *comparison* object was stiffer. The pale curves represent the force condition, and the dark curves are the stretch condition. A rightward shift of the dark curve indicates an increase in stiffness perception due to the skin stretch, whereas a leftward shift indicates a decrease. ***b***, The real (blue shades) and predicted (red shades) average PSE of all 38 participants for the force and stretch conditions. The bars show the average effects, and the black error bars are the 95% confidence intervals. ***c***, The results of all 38 participants. The gray stars show each participant’s predicted Δ*PSE* relative to their real one. The black line shows the regression of the predicted Δ*PSE*s against the real Δ*PSE*s. The dashed black lines show the zero on the abscissa and ordinate. The gray dashed line shows the ideal model, that is, a model with an intercept of zero and a slope of one.

Figure 8*a* shows an example of a participant’s real and predicted psychometric curves. The red (predicted) curves were created using the *Maximum Penetration Model’s* predictions for the participant. Figure 8*a* shows that the model was not able to predict the perceptual augmentation caused by the artificial skin stretch for this participant. Figure 8*b* shows the real and predicted average PSE values of the participants in each the force and stretch conditions. As seen by comparing the pale red and blue bars, representing the force condition, a near-zero PSE value was predicted relatively well. In the stretch condition, on the other hand, there was a real average PSE value of approximately 20 [N/m] (dark blue bar), whereas the predicted PSE value for this condition was near zero. This demonstrates the model’s lack of ability to predict the participants’ average augmentation effect. The regression of the Δ*PSE*_*predicted*_ against the Δ*PSE*_*real*_ for all the participants (Fig. 8*c*), shows that the model was not able to predict the perceptual augmentation for all the participants.

The results of all the simple models are shown in Table 1. Most of the models had relatively low accuracy. The accuracy of the logistic regression model is presented as an average and standard deviation due to the *k*-fold validation. The regression parameters for all of the simple models revealed that none were able to predict the perceptual augmentation caused by the artificial skin stretch. Additionally, from the average *PSE* *Error*s across participants, we found that none of the simple models were able to predict the PSE of the stretch condition, and most had large errors also in the force condition. Similarly, most of the simple models had large *JND* *Error*s. The simple model displaying the lowest errors was the *Maximum Penetration Model*, which predicted the force condition and the shapes of the curves well but was unable to predict the skin stretch perceptual augmentation.

**Table 1.** Model prediction performance

		Regression (Predicted Δ*PSE* Against Real Δ*PSE*)			PSE Error [N/m]		JND [N/m]			
		Intercept [N/m]	Slope [au]	*p*- value	Force (Real = 0.16)	Stretch (Real = 20.64)	Force (Real = 12.13)		Stretch (Real = 14.95)	
Model	Accuracy						Predicted	Error	Predicted	Error
Maximum penetration	69.51	−2.67	−0.15	0.13	−1.84	24.35	16.97	−4.84	18.93	−3.97
Maximum velocity	54.15	11.5	−0.06	0.96	−0.51	9.67	73.20	−61.07	55.26	−40.32
Average velocity	50.19	−16.95	−3.46	0.58	−79.10	29.16	2447.30	−2435.13	−133.31	148.25
Maximum grip force	59.18	−5.75	−0.18	0.86	11.41	41.33	136.58	−124.45	94.65	−79.70
Logistic regression	64.54 ± 0.03	6.85	−0.30	0.77	−70.57	−50.81	48.78	−36.66	61.23	−46.28

**Table 2.** Contribution of each action signal

		Regression (Predicted Δ*PSE* Against Real Δ*PSE*)			PSE Error [N/m]		JND [N/m]			
		Intercept [N/m]	Slope [au]	*p*-value	Force (Real = 0.16)	Stretch (Real = 20.64)	Force (Real = 12.13)		Stretch (Real = 14.95)	
Training Signals	Accuracy						Predicted	Error	Predicted	Error
All four signals	77.91 ± 0.003	12.53 ± 1.02	0.30 ± 0.03	0.06 ± 0.01	0.50 ± 0.27	2.17 ± 1.31	9.54 ± 0.35	2.59 ± 0.35	14.11 ± 0.36	0.84 ± 0.36
No grip force	75.54 ± 0.002	0.92 ± 0.77	−0.13 ± 0.03	0.10 ± 0.09	−4.07 ± 0.62	18.13 ± 0.56	10.71 ± 0.25	1.41 ± 0.25	10.34 ± 0.39	4.61 ± 0.39
Only grip force	**68.48 ± 0.001**	**16.89 ± 1.97**	**1.11 ± 0.15**	**0.02 ± 0.04**	**−13.10 ± 2.34**	**−32.33 ± 2.28**	**48.57 ± 6.51**	**−36.44 ± 6.51**	**41.04 ± 1.86**	**−26.09 ± 1.86**
No position	**76.14 ± 0.001**	**12.27 ± 0.86**	**0.33 ± 0.03**	**0.03 ± 0.02**	**0.97 ± 1.20**	**2.38 ± 1.27**	**13.54 ± 0.38**	**−1.41 ± 0.38**	**15.57 ± 0.20**	**−0.62 ± 0.20**
No velocity	**77.89 ± 0.002**	**11.34 ± 0.46**	**0.35 ± 0.01**	**0.04 ± 0.01**	**0.04 ± 0.42**	**2.02 ± 0.40**	**9.55 ± 0.13**	**2.58 ± 0.13**	**14.35 ± 0.24**	**0.60 ± 0.24**
No acceleration	**76.67 ± 0.001**	**12.56 ± 0.80**	**0.43 ± 0.03**	**0.02 ± 0.01**	**0.60 ± 0.42**	**−0.32 ± 0.37**	**11.51 ± 0.13**	**0.62 ± 0.13**	**16.32 ± 0.22**	**−1.37 ± 0.22**
Grip force and position	**76.25 ± 0.003**	**13.55 ± 0.57**	**0.48 ± 0.03**	**0.02 ± 0.01**	**−0.02 ± 0.13**	**−2.90 ± 0.89**	**12.78 ± 0.42**	**−0.66 ± 0.42**	**17.55 ± 0.41**	**−2.61 ± 0.41**

Bold is significant regression.

Taken together, the different metrics demonstrate the lack of our simple models’ abilities to predict the participants’ perceptual responses. The regression coefficients show the inability of the models to predict the perceptual augmentation caused by the artificial stretch. The *PSE* *Error* and *JND* *Error* metrics show the inability to predict curves of similar shape to those of the participants. The *PSE* *Error* also further conveys the lack of ability to predict the perceptual augmentation. Hence, our simple models were unable to predict individual participant effects, average effects, and trends. We note that the fact that our simple models did not succeed in predicting perception from our computed metrics does not guarantee that no metric or simple model can yield a successful prediction. Rather, this demonstrates that these metrics are not suitable for such prediction, and indicates that this prediction task may be more complex than what can be captured using only simple metrics and simple models. We therefore continued our exploration using the artificial neural network.

### What is the contribution of each action signal to the prediction?

We next aimed to explore the role of each of the four action signals in the prediction using our neural network. Furthermore, we wondered if some action signals contributed more to the performance and if others may actually not be necessary. To investigate this, we continued with Dataset 1, removed each action signal in turn, and evaluated the effect on each of our metrics.

**Figure 9.**
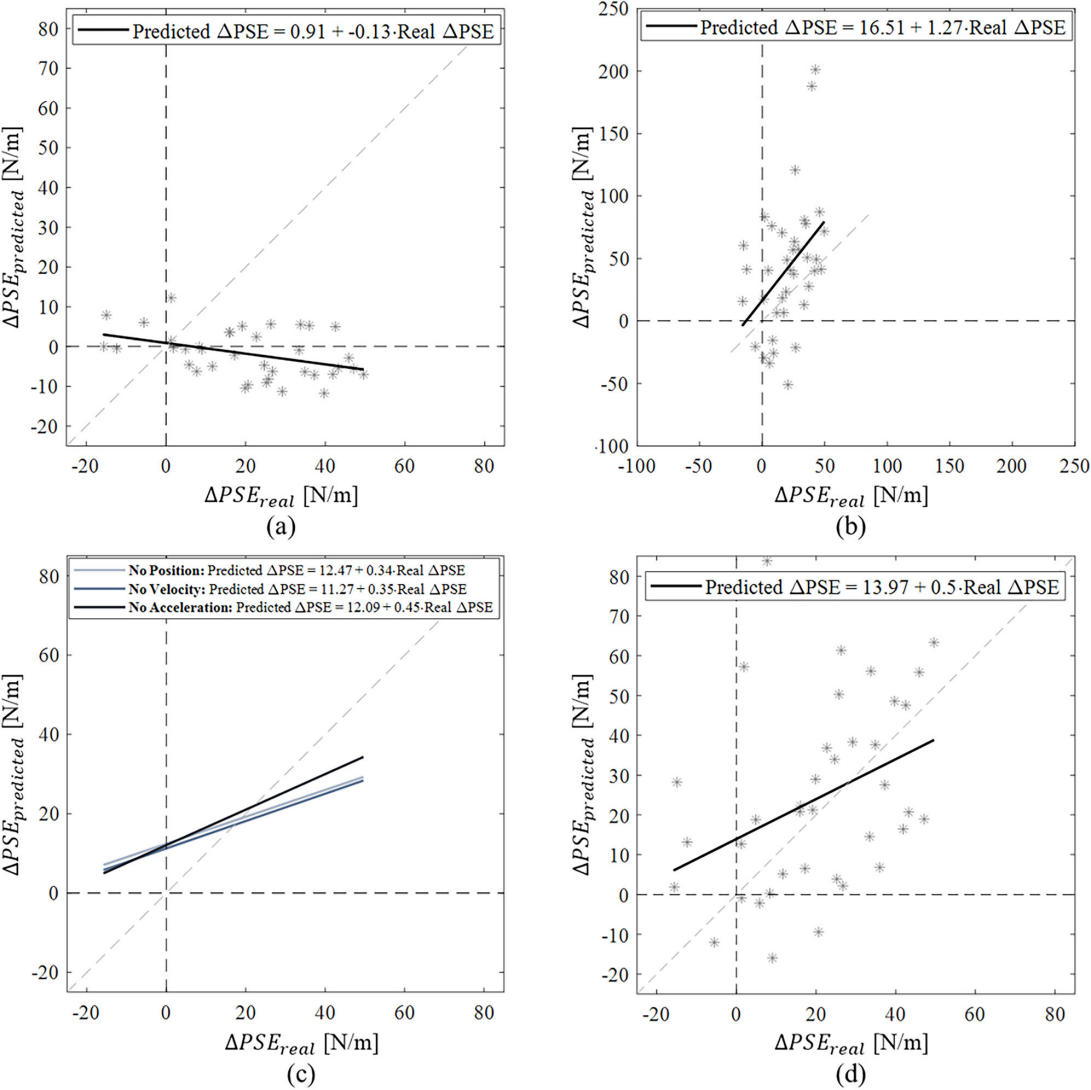
Contribution of action signals to the prediction. ***a***, No grip force. ***b***, Only grip force. ***c***, Each motion signal removed in turn. Pale blue is no position; the medium blue is no velocity; and the dark blue is no acceleration. ***d***, Position and grip force (i.e., no velocity and acceleration). Each graph shows the results for one of the five runs, the average and standard deviation of all five runs are reported in Table 2. The dashed black lines show the zero on the abscissa and ordinate. The gray dashed line shows the ideal model, that is, a model with an intercept of zero and a slope of one. In ***a***, ***b***, and ***d***, the gray stars show each participant’s predicted Δ*PSE* relative to their real one.

We first removed the grip force and found that this eliminated the ability of the network to predict the augmentation effect caused by the skin stretch (Fig. 9*a*). This indicates that, due to the stretch, participants changed their grip force to be more similar to interactions with stiffer objects. The full effect of removing the grip force can be seen in Table 2. The regression parameters and skin stretch *PSE* *Error* reflect the lack of ability to predict the perceptual augmentation without the grip force.

After discovering the importance of the grip force for predicting the augmentation effect, we wondered if this signal might be able to predict participants’ perception on its own. As shown in Figure 9*b*, this led to a wide range of over and under-estimations of participants’ stiffness perception. Examining the *PSE* *Error*s and *JND* *Error*s in Table 2 shows high errors relative to the other conditions. This reflects the inability to predict psychometric curves of similar shape to those of the participants.

The effect of removing either the grip force, or all three motion signals (position, velocity, and acceleration), led us to conclude that the grip force is necessary for predicting the augmentation effect, but that at least some of the motion signals are needed to predict the shapes of the curves. To test the contribution of each motion signal, we removed each in turn (Fig. 9*c*). We found that this did not eliminate the network’s ability to predict the augmentation effect or curves of similar shapes to those of the participants. In fact, Table 2 shows that this improved or didn’t decrease the intercept, and improved the slope, of the regression. Additionally, in all three cases, the regressions were significant. Furthermore, the *PSE* *Error*s and *JND* *Error*s indicate that the prediction of the shape of the curves was comparable to when using all four signals.

Removing the velocity improved the intercept and the slope, and removing the acceleration led to an even larger slope. We therefore tested the effect of removing both and using only the position and grip force (Fig. 9*d*). As shown in Table 2, the intercept was less good, but this combination obtained the best slope and significant regression. The *PSE* *Error* values were slightly higher in the stretch condition, but lower in the force condition, than those achieved with all four signals. Similarly, the *JND* *Error* in the force condition was better in this case, and less good in the stretch condition. These results lead us to believe that it is sufficient to use only the grip force and position to predict the participants’ perceptual responses. Despite the improvement, it is still important to note that our model is not ideal. The network continues to exhibit errors in predicting the effect sizes, however, it successfully predicted a significant trend of increased predicted augmentation for increased real augmentation. As we found that using the position and grip force led to similar or better results as all four signals, we continued with these two signals for the next part of the study.

### Can the network learn the difference in the magnitudes of the perceptual effects caused by positive and negative artificial skin stretch?

After observing that the trend of increased stiffness perception could be predicted from action signals, we explored the negative stretch session. Farajian et al. (2023) showed that similar to the positive stretch, the negative stretch also led to an average increase in stiffness perception. However, this effect was generally smaller than that caused by the positive stretch (although there were participants who exhibited the opposite). We tested the network’s ability to predict this difference on average and within each participant. That is, can the network predict the average increases in stiffness perception caused by the two directions of skin stretch, and can it predict the direction of stretch that leads to a larger perceptual effect for each participant?

**Figure 10.**
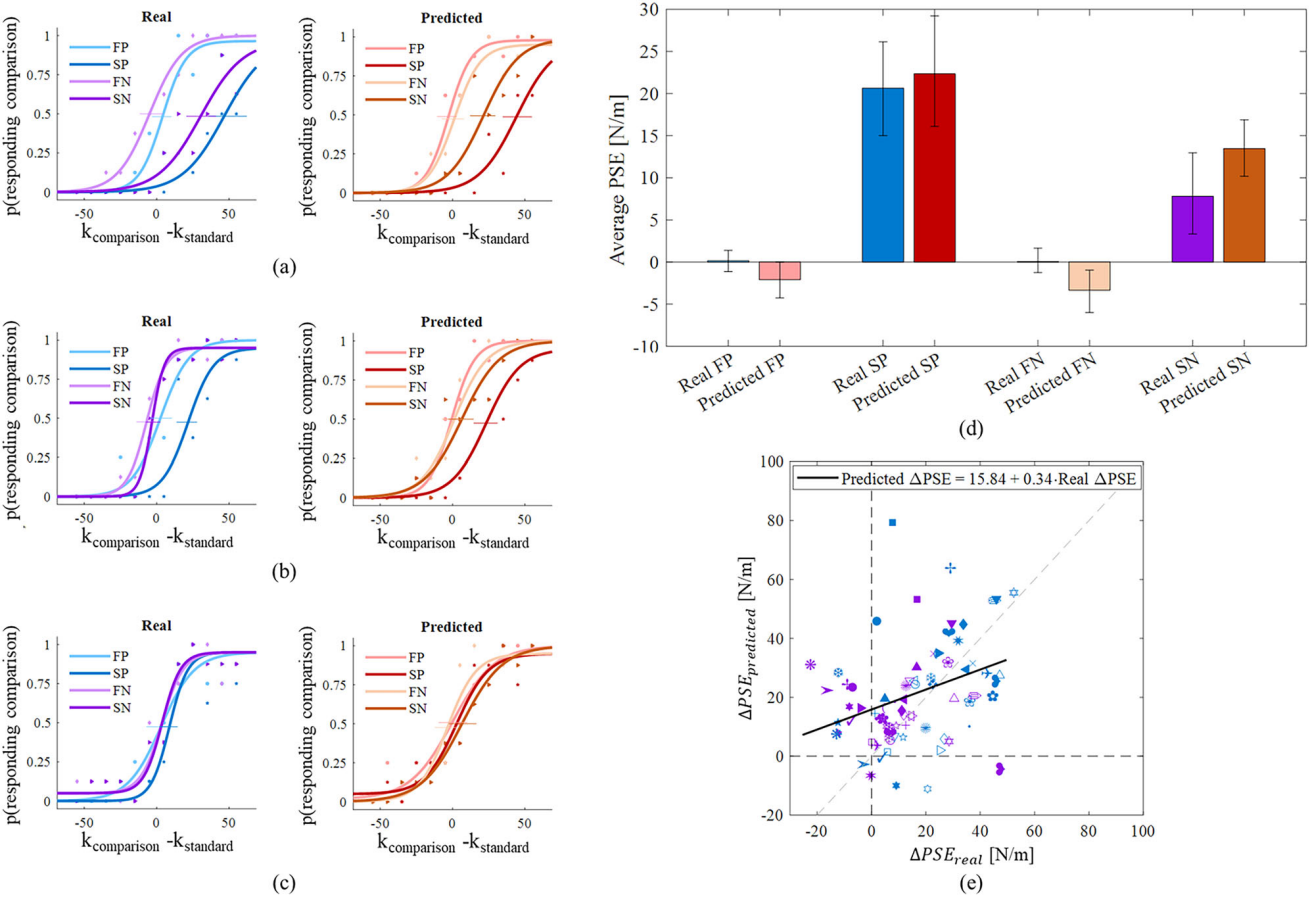
Positive and negative stretch. ***a–c***, Examples of real and predicted psychometric curves. The abscissa is the difference between the stiffness levels of the two virtual objects, and the ordinate is the probability of choosing that *comparison* was stiffer. The blue and purple curves were created using the participants’ real responses, whereas the red and orange curves were created from the network’s predictions. The blue and red represent the positive stretch session (P), and purple and orange are the negative stretch session (N). The pale curves represent the force conditions (F), and the dark curves are the stretch conditions (S). ***d***, The average real and predicted PSE for each condition for one of the five runs, where the colors correspond to those of the psychometric curves. The error bars show the 95% confidence intervals. ***e***, The predicted Δ*PSE* as a function of the real Δ*PSE* for one of the five runs. Each symbol represents a participant, where the blue are the positive stretch session, and the purple are the negative stretch session. The regression line is shown in black. The dashed black lines show the zero on the abscissa and ordinate. The gray dashed line shows the ideal model – a model with an intercept of zero and a slope of one.

To test this, we used Dataset 2, which included the two force conditions, the positive stretch condition, and the negative stretch condition. Figure 10*a–c* show examples of psychometric curves for some of the participants whose perceptual effects were predicted well by the network. In all three examples, the curves for the two force conditions were similar, as expected. In Figure 10*a–b*, the effect of the positive stretch was larger than that of the negative stretch, where in Figure 10*b*, the effect of the negative stretch was near zero. Figure 10*c* shows an example of a participant who was, in large, not affected by the artificial stretch.

Figure 10*d* shows the average results of all the participants. The bars show the real and predicted increase in perceived stiffness for each of the four conditions. The pale-colored bars show that the real and predicted augmentation effects for the force conditions were near zero. The dark-colored bars represent the stretch conditions, where the dark blue and red are the real and predicted positive stretch augmentations. The dark purple and orange are the real and predicted negative stretch condition. Both the real and predicted negative stretch augmentation effects are lower than those of the positive session. This indicates the ability of the network to predict the average trends shown by the participants. That is, near zero augmentation effects for both force conditions, and a higher increase in stiffness perception in the positive session relative to the negative session.

Figure 10*e* shows the regression of the Δ*PSE*_*predicted*_ against the Δ*PSE*_*real*_ for all the participants. Each symbol represents a participant, each of which has two points - blue for the positive session and purple for the negative session. As shown, there is dispersion of the data, and the regression parameters are not ideal. However, the same general trend of increased predicted augmentation for increased real augmentation continues to hold. That is, these results are consistent with the previous parts of our work, showing that the effect sizes per participant cannot be predicted by our network, but the general trend can and is statistically significant. The full results of the prediction are shown in Table 3.

**Table 3.** Positive and negative skin stretch

	Regression (Predicted Δ*PSE* Against Real Δ*PSE*)			PSE Error [N/m]			JND Error [N/m]				
Accuracy	Intercept [N/m]	Slope [au]	*p*-value	Force Positive (Real = 0.16)	Stretch Positive (Real = 20.64)	Force negative (Real = 0.06)	Stretch Negative (Real = 7.81)	Force positive (Real = 12.13)	Stretch Positive (Real = 14.95)	Force negative (Real = 12.38)	Stretch Negative (Real = 14.64)
**75.58 ± 0.001**	**16.01 ± 0.51**	**0.31 ± 0.03**	**0.007 ± 0.007**	**2.11 ± 0.37**	**−1.73 ± 0.70**	**3.20 ± 0.18**	**−5.40 ± 0.34**	**−1.04 ± 0.14**	**−2.10 ± 0.27**	**−1.54 ± 0.22**	**−1.64 ± 0.33**

Bold is significant regression.

In this part of the work, we aimed to assess the ability of the network to predict the difference in the perceptual augmentation caused by the positive and negative stretch. Figure 10*d* demonstrated the ability of the network to do so on average. Figure 10*e* can shed light on the ability to do so per participant. By comparing the blue and purple symbols for each participant, we can evaluate if the network was able to predict which stretch direction would have a larger perceptual effect on that participant in absolute value. For most participants, the positive stretch had a larger effect on the perceived stiffness than the negative stretch, however, there were also participants who showed the opposite trend. We found that the network was able to correctly predict which stretch direction would lead to a larger effect, and which to a smaller effect, for 25 out of 38 participants, which is approximately 2/3. These results demonstrate the ability of the network to predict a general trend for the participants on average, and within many of the participants.

## Discussion

We aimed to predict the participants’ perception from their recorded action signals (position, velocity, acceleration, and grip force). We utilized data recorded in a stiffness discrimination task (Farajian et al., 2023) that was comprised of force conditions and artificial skin stretch conditions. We assessed the ability of simple models and artificial neural networks to predict participants’ stiffness perception in each condition separately and explored the success in predicting the perceptual augmentation caused by the artificial stretch. We found that artificial neural networks, but not simple models, could predict participants’ perceptual augmentation to an extent from their action signals alone.

Our simple models could not predict the perceptual augmentation caused by the skin stretch. Furthermore, the simple models were generally not able to predict the shapes of the participants’ psychometric curves. This corresponds to the issue raised in Carey (2001), Smeets and Brenner (2006) and Smeets et al. (2002), who discuss the importance of selecting appropriate measures to assess the effect of perceptual illusions on action. As the artificial skin stretch has been shown to affect grip force (Farajian et al., 2020), we included a metric computed from this signal, however, found it to be insufficient to predict the participants’ perceptual responses. Our best-performing simple model was the *Maximum Penetration Model*, however, this model did not predict the perceptual augmentation caused by the artificial stretch. The lack of ability to predict stiffness perception from the penetration distance alone corresponds to the results in Pressman et al. (2007) and Nisky et al. (2008), in which they examined models for stiffness perception in the presence of delayed force fields. Our results do not guarantee that no simple model would be able to predict participants’ perception. However, we found that the artificial neural networks yielded better prediction, indicating that simple metrics might not be sufficient. It is possible that the network learns from the richer signal containing data from the entire interaction with the object, or from the temporal relation between the samples of the signal.

Our artificial neural network was able to capture a general trend of predicted increased stiffness perception for real increased stiffness perception. Despite this, the prediction was not ideal, and the network fell short in the prediction of the precise effect size for each participant. By examining the distribution of the network errors for the different *comparison* stiffness levels, we discovered that more errors were made for higher *comparison* stiffness levels relative to the lower ones. Hypothetically, we could assume that this distribution should have been symmetrical around 85 [N/m]. This was indeed the case when examining the force trials separately. However, this was not the case when examining the stretch trials, and all the trials together. This lack of symmetry can be explained by the skin stretch trials. The fact that the network was able to predict an increase in stiffness perception due to the stretch indicates that participants’ signals may have changed to resemble those of interactions with stiffer *comparison* levels when encountering this stimulus. Therefore, the presence of the stretch would decrease the errors for lower *comparison* stiffness levels and could increase the errors for higher *comparison* levels, where the *comparison* and *standard*-with-skin-stretch signals may be more similar. Despite the apparent effect of the stretch trials, it is important to note that the asymmetry was greater for the stretch trials separately, such that when examining all the trials together, the effect of both conditions could be observed.

The fact that the perceptual augmentation trend was predicted can indicate that at least some perceptual information is present in action signals, or can be inferred from them, enabling the network to achieve this prediction. It is possible that a more precise prediction could have been achieved by a different network. We chose to use a simple LSTM network to learn the temporal relations in the action signals. By doing so, we showed that this prediction is possible; future work comparing different networks is needed to ascertain if there are superior architectures for this task. An additional factor that may have impeded the network’s ability to predict exact effect sizes per participant is the possibility of ghost forces that could have been applied by the haptic device. These forces could have affected either or both perception and action, however, are not measurable as the Phantom 1.5 only allows for recording the commanded forces.

A different hypothesis can be derived from Farajian et al. (2020), which showed that the perceptual increase caused by skin stretch is immediate, whereas the effect on grip force control is gradual. It is therefore possible that participants’ action signals from the initial interactions with the *standard* object in the stretch condition do not reflect the increase, whereas later ones do. This may have caused later probing movements to resemble those of interactions with stiffer objects, while earlier ones may not have. This could have challenged the ability of the network to learn the exact augmentation effect per participant. A potential solution to this challenge could be inputting only the final interaction into the network. When we tried to do so, we found that it did not leave enough samples to enable the network to predict the participants’ perceptual responses.

Another possibility is that the action signals we used are indicative of the perception only to an extent. Perhaps not all the perceptual information can be found in these four action signals. A further hypothesis can be that the artificial skin stretch affected perception and action to different extents, which may too have differed between participants. Previous studies have shown cases in which illusions affect both perception and action, however, to different extents (Smeets and Brenner, 1995; Franz et al., 2001; Yamagishi et al., 2001), or even cause effects in opposite directions (Haffenden and Goodale, 2000). For example, in Franz et al. (2001), they examined the Muller-Lyer and Parallel Line illusions. They found that the Muller-Lyer illusion had a larger effect on action than on perception, whereas the Parallel Line perceptual illusion was higher than the action illusion. Furthermore, in Yamagishi et al. (2001), they showed conditions under which the effect of a moving background illusion on action is three times bigger than the perceptual effect, whereas, under other conditions, the two effects are of similar magnitude. Similar to these illusions, it is possible that the skin stretch led to different magnitudes of perceptual and action effects for different participants, leading to the ability to predict a general trend, but not precise effect sizes.

Despite the inability of the network to predict each participant’s effect size, it was able to predict the average perceptual effects of the participants relatively well. This can be seen by the low *PSE Error* and *JND Error* values, indicating that the network was able to predict the shapes of participants’ psychometric curves and the perceptual augmentation well on average. This corresponds to Bi et al. (2018a), in which they trained models to perform a stiffness discrimination task of the bending stiffness of cloth. They trained the models to predict the average results of all the participants and showed a high correlation between the average perceptual scale of the participants and that predicted by the model. However, they did not examine the ability of their model to predict the perceptual effects of individual participants. Furthermore, in their work, the input to the network described the stimulus, whereas our network learned solely from participants’ action signals. Therefore, the questions addressed in their work are related to the ability of a model to perform such a task similarly to humans, whereas our work deals with the question of predicting perception from action. Our network predicted the participants’ average results well with no information about the stimuli with which the participants interacted.

After discovering that the network could predict the participants’ perception on average, and a general trend, but not precise effect sizes, we explored the contribution of each action signal to the prediction. We found that the network could not predict the perceptual augmentation caused by the stretch without the grip force. This is supported by Farajian et al. (2020) and Farajian et al. (2023), in which an increase in the grip force modulation with the load force was shown due to artificial skin stretch. However, this metric utilizes both grip force, which is a signal describing the participants’ action, and load force, which describes the stimulus, whereas we used only the grip force. Our results show that grip force can be informative about the perceptual augmentation even when it is not regressed on the load force. On the other hand, providing the model with only grip force, and no motion signal, decreased the ability of the model to predict psychometric curves with similar shapes to those of the participants. These results demonstrate the importance of the grip force for predicting participants’ stiffness perception. However, they also show that this signal is not enough on its own.

By removing each motion signal (position, velocity and acceleration) in turn, we concluded that all three signals may not be necessary. We found that providing a motion signal with the grip force signal is necessary to obtain a prediction of the perceptual augmentation and participant-like shaped curves. These curves are characterized by the fact that the more different the stiffness levels of the *standard* and *comparison* virtual objects are, the fewer mistakes are made. By removing each motion signal in turn, we learned that inputting them into the network together may actually decrease the performance. This is similar to our results in Kossowsky and Nisky (2022), in which removing specific signals improved the performance of a network. This can be due to a redundancy between them, and inputting only the necessary information can assist the network’s learning. This can be due both to providing simpler data containing only the most necessary information, as well as the reduction in the number of parameters the network needs to learn due to the smaller data. Our results indicate that, of the three motion signals we tested, the position appears to contribute more than the other two.

Lastly, we assessed the ability of the network to predict the difference between the perceptual effects of positive and negative artificial skin stretch. The positive stretch led to a larger and more consistent perceptual augmentation than the negative stretch. A possible explanation for this difference is offered by the model in Farajian et al. (2023), in which we suggest that the perceptual effect is related to non-uniform distribution of mechanoreceptors across the finger pad, and the fact that the tactile afferent neurons have preferred directions (Birznieks et al., 2001; Johansson and Flanagan, 2008). Furthermore, the different stretch directions have been shown to lead to different changes in certain aspects of grip force (Farajian et al., 2023). Our results indicate that our network was able to learn these differences on average across participants, and within many of the participants. The first is demonstrated by the ability to predict a larger average increase due to the positive stretch than that of the negative stretch. The latter is shown by the ability to predict the stretch direction which would lead to the larger perceptual effect for many of the participants.

In this work, we explored what simple models and artificial neural networks can learn about the participants’ perception from their action signals. We learned that our neural network can predict perceptual trends from action signals alone, indicating that at least some perceptual information is present in the action signals. However, our network fell short in the prediction of the effect sizes and direction. Further work is needed to ascertain if this is due to the architecture of the network or amount of data, or rather to the perceptual information present in the action signals. We found that the information in the grip force signal is necessary for predicting the perceptual augmentation caused by the skin stretch, and motion signals are necessary for predicting curves with similar shapes to those describing human perception. This exploration sheds light on what perceptual aspects can be learned from different action signals. This work can also potentially be relevant for robotic devices in which haptic stimuli are presented to the user. In these cases, one can record action signals, however, the perceptual experiments required to measure the effects of different stimuli are long. Having trained networks that can predict perceptual effects from action signals can allow for assessing the perceptual effects of haptic stimuli in a more efficient manner. However, further studies are required to further evaluate the feasibility of such an application.

## Synthesis

Reviewing Editor: David Franklin, Technische Universitat Munchen Decisions are customarily a result of the Reviewing Editor and the peer reviewers coming together and discussing their recommendations until a consensus is reached. When revisions are invited, a fact-based synthesis statement explaining their decision and outlining what is needed to prepare a revision will be listed below. The following reviewer(s) agreed to reveal their identity: Frida Torell.

Your manuscript has been re-reviewed by the two original reviewers. They both appreciate the revisions that you have done. We believe that this work provides new insight into the use of neural networks to predict action signals, and demonstrate that perceptual judgements can be directly inferred from motion signals. However, we have a few minor suggestions that could improve the final version of the manuscript. Please revise the manuscript, taking these points into consideration.

Specific comments from the reviewers.

Reviewer 1

Line 584 seems to contain two double blanks.

Figure 7: The skin stretch trials do not present the 85 N/m pattern, there the value is rather 100-105 N/m. The strong pattern in the force trials affects the figure where all trials are combined. Why is this not mentioned in the text? Nor discussed in the Discussion?

Reviewer 2

The authors have satisfactorily addressed my concerns, and I find this to be a very creative study. I have only a few suggestions regarding the introduction to help improve its structure and better motivate the research questions.

In the title and throughout the manuscript, the authors use the generic term "models," which can lead to confusion, as ANNs are also considered models. It appears that the authors are specifically referring to models that rely on direct motion signals, as opposed to ANNs, which learn the features automatically from data through training. To avoid ambiguity, the authors should clearly specify what "models" refer to and emphasize the distinction between these models and ANNs.

Lines 38-54: The authors focus on discussing debates on whether perception and action are associated or dissociated, which leads the reader to expect a contribution toward resolving this issue. However, their actual focus is on whether one signal can be decoded from the other under the assumption that the association exists. In other words, the study's findings cannot address this broader debate, as the authors use a specific illusion that already supports an associative relationship. If a different illusion were used (one that does not support association), it is unclear if the same decoding would be successful. A more appropriate introduction would briefly acknowledge the broader debate and then shift the focus to studies investigating the inferability of one signal from another when an association is present, ensuring that the initial motivation aligns with the specific research focus.

Lines 86-106 seem to suggest that models based on action signal metrics and artificial neural networks represent fundamentally different modeling approaches. However, this distinction is not entirely accurate. The reason is that unless a specific assumption about the form of the mapping (e.g., linear vs. non-linear) is made, ANNs and other models are not inherently different types of approaches.

Artificial neural networks (ANNs) are universal function approximators, meaning they can theoretically represent a wide range of mappings, both linear and non-linear. Therefore, the primary advantage of ANNs is not that they constitute a fundamentally different class of model but rather that they provide more flexibility and statistical power to learn complex patterns directly from data without requiring predefined feature engineering.

In other words, models using hand-crafted features and ANNs can theoretically capture the same relationships given sufficient capacity and data. The main distinction lies in ANNs' ability to automatically learn these mappings from raw inputs without specifying the features a priori. Thus, framing these as entirely distinct modeling paradigms is misleading unless there is a specific assumption about the nature of the mapping (e.g., linearity or time dependency in the case of LSTMs).

Lines 108-110: The main research question is "whether perceptual information can be inferred from motion signals." To explore this, they pose two specific sub-questions: "what aspects of perception can be learned from each signal" and "which perceptual trends can be predicted." However, these two questions are not clearly defined and create confusion for the reader.

For the first sub-question, it is unclear what the authors mean by "different action signals" or which types of perceptual aspects they are referring to. Similarly, the second sub-question, "which perceptual trends can be predicted," is overly broad. The term "perceptual trends" is ambiguous and could refer to anything from dynamic changes in perception to long-term temporal patterns or spatial variations. Without a clear explanation or examples in the introduction, these sub-questions lack motivation and do not provide a solid rationale for the study.

To improve clarity and coherence, the authors should clearly align the two sub-questions with the primary research goal of inferring perceptual information from motion signals. Additionally, providing concrete definitions and examples of specific perceptual features and trends that are relevant to motion signals, along with a review of existing gaps in the literature, would better support and motivate these research questions.

## Author Response

Dear editor and reviewers, In the following, we provide a point-by-point response to the reviewers' comments. We took all the comments onboard, and have addressed each of the reviewer's comments below. Throughout the reply, we use the following color-coding: − Original comments by the reviewers - black regular font − Our responses - black italic font − Citations from the revised paper - "red bold font in quotation marks" Thank you for your comments and consideration of our work, Hanna Kossowsky Lev and Prof. Ilana Nisky Reviewer 1: 1. Line 584 seems to contain two double blanks. We thank the reviewer for the attention to detail and have checked this. Surprisingly, we discovered that there are not double blanks, and that this is due to the formatting of the LaTeX document. Due to the additions made following this review, the spacing has changed and there is no longer appears to be double blanks in the mentioned line. 2. Figure 7: The skin stretch trials do not present the 85 N/m pattern, there the value is rather 100- 105 N/m. The strong pattern in the force trials affects the figure where all trials are combined. Why is this not mentioned in the text? Nor discussed in the Discussion? This was indeed not adequately discussed. We have added the following (underlined) to the discussion about the figure in the Results: "We also analyzed the distribution of errors in the network's predictions relative to the participants' responses (the true labels) for the different comparison stiffness levels (Fig. 7(a-c)). An examination of Fig. 7(c) (stretch trials only) reveals an asymmetrical distribution, with a larger percentage of errors for higher comparison stiffness levels. This can be due to the artificial skin stretch. The fact that the network could predict a general trend of increased stiffness perception due to the stretch indicates that participants' action signals may resemble those of interactions with higher stiffness levels. Therefore, the skin stretch may cause the standard signals to resemble those of interactions with higher stiffness values than 85 [N/m] (the standard stiffness level). Therefore, for comparison stiffness levels below 85 [N/m], the skin stretch could increase the chance that the network chose standard as stiffer, which is likely the same response as the participant, as the skin stretch increased the perceived stiffness. For comparison stiffness levels higher than 85 [N/m], there may be more errors due to the action signal not reflecting the precise perceptual increase experienced by the participant. This can cause the network either to wrongly predict that participants perceived the standard object as stiffer, when they did not, or visa versa. Fig. 7(b) shows the force only trials, in which a more symmetrical distribution of the errors can be observed, as expected. Fig. 7(a) shows all the trials together, and is, therefore, a combination of the two described distributions of errors. On the one hand, the distribution is asymmetrical due to the influence of the stretch trials. On the other hand, there is a peak around 85 [N/m], due to the force trials." And to the Discussion: "By examining the distribution of the network errors for the different comparison stiffness levels, we discovered that more errors were made for higher comparison stiffness levels relative to the lower ones. Hypothetically, we could assume that this distribution should have been symmetrical around 85 [N/m]. This was indeed the case when examining the force trials separately. However, this was not the case when examining the stretch trials, and all the trials together. This lack of symmetry can be explained by the skin stretch trials. The fact that the network was able to predict an increase in stiffness perception due to the stretch indicates that participants' signals may have changed to resemble those of interactions with stiffer comparison levels when encountering this stimulus. Therefore, the presence of the stretch would decrease the errors for lower comparison stiffness levels, and could increase the errors for higher comparison levels, where the comparison and standard-with-skin-stretch signals may be more similar. Despite the apparent effect of the stretch trials, it is important to note that the asymmetry was greater for the stretch trials separately, such that when examining all the trials together, the effect of both conditions could be observed." Reviewer 2: 1. In the title and throughout the manuscript, the authors use the generic term "models," which can lead to confusion, as ANNs are also considered models. It appears that the authors are specifically referring to models that rely on direct motion signals, as opposed to ANNs, which learn the features automatically from data through training. To avoid ambiguity, the authors should clearly specify what "models" refer to and emphasize the distinction between these models and ANNs. We thank the reviewer for raising this inaccuracy. In our work, we used "simple models", which received specific action metrics, and ANNs, which received action signals themselves, and learned features during the training process. As both are in fact models, we adjusted the entire paper such that we use the term "models" when referring to both. When referring to the models that received specific action metrics, we use the term "simple models". When referring specifically to our ANN, we use artificial neural network, or neural network. These changes have been made throughout the entire paper and are therefore not quoted here. However, this did include changing the title from: "Can Information About Stiffness Perception be Inferred from Action Signals Using Models and Artificial Neural Networks?" to "Can Information About Stiffness Perception be Inferred from Action Signals Using Models?", such that now the word "models" refers to both simple models and ANNs. 2. Lines 38-54: The authors focus on discussing debates on whether perception and action are associated or dissociated, which leads the reader to expect a contribution toward resolving this issue. However, their actual focus is on whether one signal can be decoded from the other under the assumption that the association exists. In other words, the study's findings cannot address this broader debate, as the authors use a specific illusion that already supports an associative relationship. If a different illusion were used (one that does not support association), it is unclear if the same decoding would be successful. A more appropriate introduction would briefly acknowledge the broader debate and then shift the focus to studies investigating the inferability of one signal from another when an association is present, ensuring that the initial motivation aligns with the specific research focus. Thank you. We shortened the debate on the association or dissociation between perception and action, and have clarified that this work focuses on the case where there is an association. "We use haptic feedback to form our perception and control actions, such as our movements and forces. The literature is ambivalent about the association or dissociation between the processing of sensory information for perception and action, with evidence in each direction (Leib et al., 2015; Bruno, 2001; Post et al., 2003; Ganel and Goodale, 2003, 2014; Goodale and Milner, 1992; Goodale and Humphrey, 1998; Flanagan and Beltzner, 2000; Franz et al., 2000; Haffenden et al., 2001; Grandy and Westwood, 2006; Platkiewicz and Hayward, 2014; Bi et al., 2018b). Many studies use perceptual illusions to explore this question. For example, the Ebbinghaus illusion was originally shown to affect size perception, but not grip aperture before object grasping (Aglioti et al., 1995). Later evidence suggested that this illusion affects both perception and action, but to different degrees (Franz et al., 2001). The debate about the association or dissociation remains unresolved. In this paper, we examine a case where perception and action appear associated and ask whether action is indicative of perception in this case." Furthermore, we have added the following paragraph to the Introduction, focusing on previous works modeling perception. These works however, have used information about the stimuli to model perception, whereas we ask if perception can be inferred from action information alone. "Previous works have used models to explain perception. For example, delays in force feedback affect stiffness perception and (Pressman et al., 2006) modeled this using force and position information. Such models can also explain how altering an elastic object's boundary affects stiffness perception (Pressman et al., 2011). Additionally, (Metzger et al., 2018; Kossowsky et al., 2021) studied stiffness perception when the force field stiffness varies between interactions. They found that participants formed their perception by computing a weighted average of the different stiffness levels. These works, however, included information about the stimuli (force or stiffness), whereas we aim to predict participants' perception using only their actions. Several visual illusions were also shown to affect both size perception and grasping, with a correlation between the effects (Franz et al., 2001). However, they did not use participants' actions to predict perceptual responses on a trial-by-trial basis, which, to our knowledge, remains unexplored. Additionally, these works did not employ machine learning models to learn perceptual information from action signals." 3. Lines 86-106 seem to suggest that models based on action signal metrics and artificial neural networks represent fundamentally different modeling approaches. However, this distinction is not entirely accurate. The reason is that unless a specific assumption about the form of the mapping (e.g., linear vs. non-linear) is made, ANNs and other models are not inherently different types of approaches. Artificial neural networks (ANNs) are universal function approximators, meaning they can theoretically represent a wide range of mappings, both linear and non-linear. Therefore, the primary advantage of ANNs is not that they constitute a fundamentally different class of model but rather that they provide more flexibility and statistical power to learn complex patterns directly from data without requiring predefined feature engineering. In other words, models using hand-crafted features and ANNs can theoretically capture the same relationships given sufficient capacity and data. The main distinction lies in ANNs' ability to automatically learn these mappings from raw inputs without specifying the features a priori. Thus, framing these as entirely distinct modeling paradigms is misleading unless there is a specific assumption about the nature of the mapping (e.g., linearity or time dependency in the case of LSTMs). We agree and thank the reviewer for raising this lack of clarity in the paper. We have adjusted the beginning of this last paragraph in the Introduction as follows: "We used data recorded in a stiffness discrimination task in which participants interacted with pairs of virtual objects that applied force and artificial skin stretch and determined which they perceived as stiffer (Farajian et al., 2023). We designed models to receive action information as input and output participants' predicted perception. We tested both simple models, which received metrics derived from action signals, and artificial neural networks, which can learn complex features, as well as non-linear, and potentially temporal, relationships from the data." Additionally, in the Methods have clarified: "We aimed to explore what artificial neural networks could learn about stiffness perception from participants' action signals. We chose to use artificial neural networks due to their statistical power to learn complex patterns directly from data without requiring predefined feature engineering, as well as their ability to capture non-linear, and temporal (when using Recurrent Neural Networks) patterns in the data." 4. Lines 108-110: The main research question is "whether perceptual information can be inferred from motion signals." To explore this, they pose two specific sub-questions: "what aspects of perception can be learned from each signal" and "which perceptual trends can be predicted." However, these two questions are not clearly defined and create confusion for the reader. For the first sub-question, it is unclear what the authors mean by "different action signals" or which types of perceptual aspects they are referring to. Similarly, the second sub-question, "which perceptual trends can be predicted," is overly broad. The term "perceptual trends" is ambiguous and could refer to anything from dynamic changes in perception to long-term temporal patterns or spatial variations. Without a clear explanation or examples in the introduction, these sub-questions lack motivation and do not provide a solid rationale for the study. To improve clarity and coherence, the authors should clearly align the two sub-questions with the primary research goal of inferring perceptual information from motion signals. Additionally, providing concrete definitions and examples of specific perceptual features and trends that are relevant to motion signals, along with a review of existing gaps in the literature, would better support and motivate these research questions. Thank you for raising this lack of clarity on our part. When referring to aspects of perception: we examined the ability of the models to predict the perceptual augmentation caused by the stretch, and their ability to predict human-like perception by assessing their ability to predict psychometric curves of similar shapes to those describing human perception. We additionally give examples of our different action signals. We have added the following to the last paragraph in the Introduction (the paragraph discussed in this comment): "We assessed if our models could predict the increase in stiffness perception caused by the skin stretch. Beyond analyzing perceptual biases, we can also evaluate participants' decision-making processes. As the difference between the two virtual objects' stiffness levels increases, humans generally make fewer mistakes. We tested our models' abilities to predict this behavior. By inputting different combinations of action (motion and grip force) signals, we studied their roles in predicting the perceptual augmentation, and human-like perception." When referring to perceptual trends, we meant the difference in the magnitude of the effect of artificial skin stretch in the same, and the opposite, direction as the force field. We have attempted to fix this by first explaining about the two directions of stretch when introducing the skin stretch perceptual illusion in the Introduction: "...When applied with force feedback, artificial skin stretch creates the perceptual illusion of increased stiffness perception (Quek et al., 2014; Farajian et al., 2020). Artificial skin stretch in both the same, and the opposite, direction as force feedback increases stiffness perception, but the effect is stronger when the stretch is applied in the same direction (Farajian et al., 2023)..." And then in the paragraph in question in this comment have added: "Additionally, we evaluated a model's ability to predict the difference in the magnitude of the augmentation effect caused by skin stretch in the same, and the opposite, direction as the force feedback."

## References

[B1] Aglioti S, DeSouza JF, Goodale MA (1995) Size-contrast illusions deceive the eye but not the hand. Curr Biol 5:679–685. 10.1016/S0960-9822(95)00133-3 7552179

[B2] Bi W, Jin P, Nienborg H, Xiao B (2018a) Estimating mechanical properties of cloth from videos using dense motion trajectories: human psychophysics and machine learning. J Vis 18:12. 10.1167/18.5.12 29904787

[B3] Bi W, Newport J, Xiao B (2018b) Interaction between static visual cues and force-feedback on the perception of mass of virtual objects. In: *Proceedings of the 15th ACM symposium on applied perception*, pp 1–5.

[B4] Birznieks I, Jenmalm P, Goodwin AW, Johansson RS (2001) Encoding of direction of fingertip forces by human tactile afferents. J Neurosci 21:8222–8237. 10.1523/JNEUROSCI.21-20-08222.2001 11588194 PMC6763843

[B5] Bruno N (2001) When does action resist visual illusions? Trends Cogn Sci 5:379–382. 10.1016/S1364-6613(00)01725-3 11520701

[B6] Carey DP (2001) Do action systems resist visual illusions? Trends Cogn Sci 5:109–113. 10.1016/S1364-6613(00)01592-8 11239810

[B9] Farajian M, Leib R, Kossowsky H, Zaidenberg T, Mussa-Ivaldi FA, Nisky I (2020) Stretching the skin immediately enhances perceived stiffness and gradually enhances the predictive control of grip force. eLife 9:e52653. ISBN: 2050-084X. 10.7554/eLife.52653 32292163 PMC7176431

[B7] Farajian M, Leib R, Kossowsky H, Nisky I (2021) Visual feedback weakens the augmentation of perceived stiffness by artificial skin stretch. IEEE Trans Haptics 14:686–691. 10.1109/TOH.2021.3052912 33465030

[B8] Farajian M, Leib R, Kossowsky H, Nisky I (2023) Direction-specific effects of artificial skin-stretch on stiffness perception and grip force control. IEEE Trans Haptics 16:836–847. 10.1109/TOH.2023.3332295 37956003

[B10] Flanagan JR, Beltzner MA (2000) Independence of perceptual and sensorimotor predictions in the size–weight illusion. Nat Neurosci 3:737–741. 10.1038/76701 10862708

[B11] Flanagan JR, Tresilian J, Wing AM (1993) Coupling of grip force and load force during arm movements with grasped objects. Neurosci Lett 152:53–56. ISBN: 0304–3940. 10.1016/0304-3940(93)90481-Y 8515879

[B12] Flanagan JR, Wing AM (1993) Modulation of grip force with load force during point-to-point arm movements. Exp Brain Res 95:131–143. 10.1007/BF00229662 8405245

[B13] Franz VH, Fahle M, Bülthoff HH, Gegenfurtner KR (2001) Effects of visual illusions on grasping. J Exp Psychol Hum Percept Perform 27:1124–1144. 10.1037/0096-1523.27.5.1124 11642699

[B14] Franz VH, Gegenfurtner KR, Buelthoff HH, Fahle M (2000) Grasping visual illusions: no evidence for a dissociation between perception and action. Psychol Sci 11:20–25. 10.1111/1467-9280.00209 11228838

[B15] Ganel T, Goodale MA (2003) Visual control of action but not perception requires analytical processing of object shape. Nature 426:664–667. 10.1038/nature02156 14668865

[B16] Ganel T, Goodale MA (2014) Variability-based garner interference for perceptual estimations but not for grasping. Exp Brain Res 232:1751–1758. 10.1007/s00221-014-3867-3 24534914

[B17] Goodale MA, Humphrey GK (1998) The objects of action and perception. Cognition 67:181–207. 10.1016/S0010-0277(98)00017-1 9735540

[B18] Goodale MA, Milner AD (1992) Separate visual pathways for perception and action. Trends Neurosci 15:20–25. 10.1016/0166-2236(92)90344-8 1374953

[B19] Grandy MS, Westwood DA (2006) Opposite perceptual and sensorimotor responses to a size-weight illusion. J Neurophysiol 95:3887–3892. 10.1152/jn.00851.2005 16641383

[B20] Haffenden AM, Goodale MA (2000) Independent effects of pictorial displays on perception and action. Vis Res 40:1597–1607. 10.1016/S0042-6989(00)00056-0 10788660

[B21] Haffenden AM, Schiff KC, Goodale MA (2001) The dissociation between perception and action in the ebbinghaus illusion: nonillusory effects of pictorial cues on grasp. Curr Biol 11:177–181. 10.1016/S0960-9822(01)00023-9 11231152

[B22] Hochreiter S, Schmidhuber J (1997) Long short-term memory. Neural Comput 9:1735–1780. 10.1162/neco.1997.9.8.1735 9377276

[B23] Ioffe S, Szegedy C (2015) “Batch normalization: accelerating deep network training by reducing internal covariate shift.” Preprint, http://arxiv.org/abs/1502.03167

[B24] Johansson R, Flanagan J (2008) Tactile sensory control of object manipulation in humans. In: *The senses: a comprehensive reference* (Gardner E, Kaas JH, eds) Vol 6, pp 67–86. San Diego: Academic Press. Available at: http://psyc.queensu.ca/~flanagan/papers/JohFla_BC_The_Senses_07.pdf

[B25] Jones LA, Tan HZ (2013) Application of psychophysical techniques to haptic research. IEEE Trans Haptics 6:268–284. 10.1109/TOH.2012.74 24808324

[B26] Kandel ER, Schwartz JH, Jessell TM, Siegelbaum S, Hudspeth AJ, Mack S (2000) *Principles of neural science*, volume 4. McGraw-hill New York.

[B27] Kingma DP, Ba J (2014) “Adam: a method for stochastic optimization.” Preprint, http://arxiv.org/abs/1412.6980

[B28] Kossowsky H, Farajian M, Milstein A, Nisky I (2021) The effect of variability in stiffness on perception and grip force adjustment. IEEE Trans Haptics 14:513–525. 10.1109/TOH.2021.3052136 33449879

[B29] Kossowsky H, Farajian M, Nisky I (2022) The effect of kinesthetic and artificial tactile noise and variability on stiffness perception. IEEE Trans Haptics 15:351–362. 10.1109/TOH.2022.3158386 35271449

[B30] Kossowsky H, Nisky I (2022) Predicting the timing of camera movements from the kinematics of instruments in robotic-assisted surgery using artificial neural networks. IEEE Trans Med Robot Bion 4:391–402. 10.1109/TMRB.2022.3156635

[B31] Leib R, Karniel A, Nisky I (2015) The effect of force feedback delay on stiffness perception and grip force modulation during tool-mediated interaction with elastic force fields. J Neurophysiol 113:3076–3089. 10.1152/jn.00229.2014 25717155 PMC4455557

[B32] Metzger A, Lezkan A, Drewing K (2018) Integration of serial sensory information in haptic perception of softness. J Exp Psychol Hum Percept Perform 44:551–565. 10.1037/xhp0000466 29022730

[B33] Nisky I, Mussa-Ivaldi FA, Karniel A (2008) A regression and boundary-crossing-based model for the perception of delayed stiffness. IEEE Trans Haptics 1:73–82. ISBN: 1939–1412. 10.1109/TOH.2008.17 27780151

[B34] Platkiewicz J, Hayward V (2014) Perception-action dissociation generalizes to the size-inertia illusion. J Neurophysiol 111:1409–1416. 10.1152/jn.00557.2013 24401709 PMC3962618

[B35] Post RB, Welch RB, Bridgeman B (2003) Perception and action: two modes of processing visual information. In: *Visual perception: the influence of H. W. Leibowitz* (Andre J, Owens DA, Harvey Jr. LO, eds), pp 143–154. American Psychological Association.

[B36] Prattichizzo D, Pacchierotti C, Rosati G (2012) Cutaneous force feedback as a sensory subtraction technique in haptics. IEEE Trans Haptics 5:289–300. 10.1109/TOH.2012.15 26964127

[B37] Pressman A, Karniel A, Mussa-Ivaldi FA (2006) Perception of delayed stiffness. In: *The first IEEE/RAS-EMBS international conference on biomedical robotics and biomechatronics, 2006. BioRob 2006*, pp 905–910. IEEE.

[B38] Pressman A, Karniel A, Mussa-Ivaldi FA (2011) How soft is that pillow? the perceptual localization of the hand and the haptic assessment of contact rigidity. J Neurosci 31:6595–6604. 10.1523/JNEUROSCI.4656-10.2011 21525300 PMC3108063

[B39] Pressman A, Welty LJ, Karniel A, Mussa-Ivaldi FA (2007) Perception of delayed stiffness. Int J Robot Res 26:1191–1203. ISBN: 0278–3649. 10.1177/0278364907082611

[B40] Quek ZF, Schorr SB, Nisky I, Okamura AM, Provancher WR (2014) Augmentation of stiffness perception with a 1-degree-of-freedom skin stretch device. IEEE Trans Hum Mach Syst 44:731–742. 10.1109/THMS.2014.2348865

[B41] Smeets JB, Brenner E (1995) Perception and action are based on the same visual information: distinction between position and velocity. J Exp Psychol Hum Percept Perform 21:19–31. 10.1037/0096-1523.21.1.19 7707030

[B42] Smeets JB, Brenner E (2006) 10 years of illusions. J Exp Psychol Hum Percept Perform 32:1501–1504. 10.1037/0096-1523.32.6.1501 17154790

[B43] Smeets JB, Brenner E, de Grave DD, Cuijpers RH (2002) Illusions in action: consequences of inconsistent processing of spatial attributes. Exp Brain Res 147:135–144. 10.1007/s00221-002-1185-7 12410328

[B44] Wenliang LK, Seitz AR (2018) Deep neural networks for modeling visual perceptual learning. J Neurosci 38:6028–6044. 10.1523/JNEUROSCI.1620-17.2018 29793979 PMC6031581

[B45] Wichmann F, Hill N (2001) The psychometric function: I. Fitting, sampling, and goodness of fit. Percept Psychophys 63:1293–1313. ISBN: 0031–5117. 10.3758/BF03194544 11800458

[B46] Witney AG, Wing A, Thonnard J -L, Smith AM (2004) The cutaneous contribution to adaptive precision grip. Trends Neurosci 27:637–643. 10.1016/j.tins.2004.08.006 15374677

[B47] Yamagishi N, Anderson SJ, Ashida H (2001) Evidence for dissociation between the perceptual and visuomotor systems in humans. Proc R Soc Lond Ser B Biol Sci 268:973–977. 10.1098/rspb.2001.1603 11370972 PMC1088696

